# LAPTM4B Confers Resistance to EGFR-TKIs by Suppressing the Proteasomal Degradation of ATP1A1 in Non-small Cell Lung Cancer

**DOI:** 10.7150/ijbs.115365

**Published:** 2026-01-01

**Authors:** Dan Liu, Minxia Liu, Dongjin Lv, Yuxiang Li, Hongjuan Guo, Bingxiao Lu, Hao Leng, Ruyu Yan, Hongtao Yu, Tomas Blom, Kecheng Zhou

**Affiliations:** 1 School of Life Sciences, Anhui Medical University, Hefei, 230032, China.; 2 Institute for Molecular Medicine Finland, Helsinki Institute of Life Science, University of Helsinki, Helsinki, 00290, Finland.; 3 Department of Biochemistry and Molecular Biology, School of Basic Medical Sciences, Anhui Medical University, Hefei, 230032, China.; 4 Department of Clinical Research, The Third Affiliated Hospital of Kunming Medical University (Tumor Hospital of Yunnan Province), Kunming, China.; 5 Department of Anatomy, Faculty of Medicine, University of Helsinki, Helsinki, 00014, Finland.; 6 Minerva Foundation Institute for Medical Research, Helsinki, 00014, Finland.

**Keywords:** EGFR-TKIs resistance, Non-small cell lung cancer, LAPTM4B, ATP1A1, Proteasomal degradation.

## Abstract

Tyrosine kinase inhibitors (TKIs) have transformed the treatment of EGFR-mutant non-small cell lung cancer (NSCLC); however, acquired resistance remains a major clinical challenge. While lysosomes have been implicated in drug resistance, their precise role in EGFR-TKI resistance remains unclear. In this study, we found that EGFR-TKI, including gefitinib and osimertinib, impaired WWP2-mediated proteasomal degradation of LAPTM4B. Through analysis of clinical tumor samples, genetic manipulation, and functional assays, we identify the lysosomal protein LAPTM4B as a key driver of EGFR-TKI resistance by enhancing EGFR phosphorylation and downstream signaling. Mechanistically, LAPTM4B interacts with ATP1A1 and facilitates its endocytosis, while simultaneously preventing its degradation by suppressing TRIM8-mediated K63-linked ubiquitination and proteasomal turnover. This stabilization of ATP1A1 enhances lysosomal acidification, ultimately promoting EGFR-TKI resistance. To identify potential therapeutic strategies, we conducted an unbiased high-content drug screen and identified compounds that suppress LAPTM4B expression. These compounds synergistically enhance the efficacy of EGFR-TKIs in NSCLC models *in vitro* and *in vivo*, with minimal toxicity. Integrative analyses of patient tissue samples, cellular models, an animal model, and cancer databases highlight the critical role of the LAPTM4B-ATP1A1-lysosomal acidification axis in EGFR-TKI resistance, providing a promising therapeutic avenue for overcoming resistance in EGFR-mutant NSCLC.

## Introduction

Non-small cell lung cancer (NSCLC) is among the most prevalent and lethal malignancies worldwide 1. Activating mutations in the epidermal growth factor receptor (EGFR) occur in approximately 40% of NSCLC cases 1,2, with exon 19 deletions (19Del) and the exon 21 L858R substitution representing the most frequent alterations 2. Targeted therapy with tyrosine kinase inhibitors (TKIs), which suppress EGFR tyrosine kinase activity and its downstream signaling pathways, is the standard treatment for NSCLC patients harboring these mutations 3. First-generation TKIs, including gefitinib and erlotinib, and the second-generation TKI afatinib, have demonstrated substantial clinical benefit in patients with EGFR-activating mutations 4,5. More recently, the third-generation inhibitor osimertinib has extended survival, particularly in patients with secondary resistance mutations acquired after prior TKI therapy 6. Despite these advances, the emergence of EGFR-TKI resistance remains inevitable, and the underlying molecular mechanisms are incompletely understood, representing a major barrier to durable therapeutic efficacy.

Lysosomes are cellular organelles responsible for the degradation and recycling of macromolecules and damaged organelles 7. Beyond this canonical role in maintaining cellular homeostasis, lysosomes have emerged as critical regulators of drug resistance through multiple mechanisms, including lysosomal drug sequestration and modulation of intracellular membrane trafficking 8. Lysosomal sequestration occurs when chemotherapeutic agents, such as doxorubicin or cisplatin, become trapped within lysosomes, preventing their delivery to target organelles and thereby diminishing therapeutic efficacy 8. Notably, a recent study demonstrated that introducing two weakly acidic hydroxyl (-OH) groups to gefitinib significantly enhanced its cytotoxicity against NSCLC cells and mitigated drug resistance 9, implicating lysosomal retention in EGFR-TKI resistance. In addition, the intracellular membrane transport system, involving endosomes and lysosomes, governs the trafficking and distribution of drug transporters, further influencing drug resistance 8. Despite growing recognition of lysosomes as a central mediator of resistance, most studies have focused chemotherapeutic resistance 8, and the contributions of lysosomal proteins to targeted therapy resistance remain poorly understood. While pioneering studies have identified factors such as SQSTM1 in EGFR-TKI resistance 9,10, the roles of other lysosomal regulators and the underlying molecular mechanisms remain largely unexplored.

Lysosomal-associated protein transmembrane 4 beta (LAPTM4B) was initially identified in hepatocellular carcinoma 11. Subsequent cohort studies have demonstrated its upregulation in multiple malignancies, including NSCLC 12, breast cancer 13, and leukemia 14, and that high LAPTM4B expression correlates with poor prognosis 12-14. Functionally, LAPTM4B promotes cancer cell proliferation 15 and metastasis 16. Furthermore, LAPTM4B has been implicated in enhancing cancer cell stemness 14,17, facilitating autophagy 18, and conferring resistance to chemotherapeutic agents 19,20. We previously reported that LAPTM4B stimulates cytoskeleton arrangement 21, promotes integrin beta1 recycling 22, counteracts ferroptosis 23, and modulates cell metabolism 24-27. Collectively, these findings underscore multifaceted role of LAPTM4B in tumorigenesis and cancer progression. Nevertheless, despite extensive evidence of its involvement in diverse oncogenic processes, the role of LAPTM4B in EGFR-TKI resistance and its underlying molecular mechanisms remain largely uncharacterized.

ATP1A1, the catalytic α subunit of the P-type Na^+^,K^+^-ATPase ion pump, is essential for establishing and maintaining electrochemical gradients across the plasma membrane 28. Dysregulated ATP1A1 expression has been reported in multiple cancers, including breast 29, gastric 30, colon 31, and non-small cell lung cancer (NSCLC) 32, where its suppression inhibits cell proliferation 30,31 and migration 31. Moreover, nanoparticle-mediated targeting of ATP1A1 has been shown to enhance the anti-tumor efficacy of doxorubicin in breast cancer models 33. Intriguingly, ATP1A1 can undergoes stimulus-induced internalization and trafficking to lysosomes under conditions such as hypoxia 34, lung injury 35, and sepsis 36. Yet, the functional significance of endosomal/lysosomal ATP1A1 and its potential contribution to cancer progression, particularly in the context of drug resistance, remains largely unexplored.

In this study, we establish a mechanistic link between LAPTM4B and the development of EGFR-TKIs resistance. By analyzing clinical tumor samples from EGFR wild-type and mutant patients, both before initial treatment and after relapse from the sample patient, alongside functional experiments using drug-resistant cell lines and genetically manipulated cell lines, we demonstrate that EGFR-TKI suppress WWP2-mediated proteasomal degradation of LAPTM4B, thereby stabilizing its expression. We further show that LAPTM4B plays a pivotal role in the acquisition of EGFR-TKI resistance. Mechanistically, LAPTM4B regulates ATP1A1 by promoting its recruitment to lysosomes and preventing TRIM8-mediated K63-linked ubiquitin-ation and degradation, which in turn enhances lysosomal acidification and drives drug resistance. To explore potential therapeutic strategies, we performed an unbiased high-content drug screen and identified compounds that selectively downregulate LAPTM4B. These LAPTM4B-suppressing agents synergistically enhance the efficacy of EGFR-TKIs in resistant cells. Importantly, in a mouse xenograft model, the combination of gefitinib and a LAPTM4B suppressor significantly reduced tumor growth with minimal toxicity.

In summary, our findings delineate a previously unrecognized lysosomal mechanism underlying EGFR-TKI resistance and establish LAPTM4B as a promising therapeutic target for overcoming drug resistance in EGFR-mutant NSCLC.

## Materials and Methods

### Human cell lines and tissue specimens

The non-small cell lung cancer (NSCLC) cell lines PC9 and HCC827 were obtained from the American Type Culture Collection (ATCC) and subjected to routine STR profiling and mycoplasma contamination testing in the present investigation.

To generate LAPTM4B knockout (KO) cells, the CRISPR/Cas9n system was employed. Cells were stably transfected with the lentiCas9-Blast plasmid to express Cas9, and subsequently transfected with the lentiGuide-Puro plasmid containing guide RNAs (sgRNAs) specifically targeting LAPTM4B, followed by puromycin selection (4 µg/ml) for a duration of 48 hours. Single clones were subsequently isolated and validated through Sanger sequencing and western blotting.

For the establishment of LAPTM4B or ATP1A1 stably overexpressing cell lines, the KO cells were transfected with pHAGE or PTSB vectors containing LAPTM4B-24-3xFlag or ATP1A1-3xFlag using a Liposomal Transfection Reagent or NanoTrans 60™ (Biogenetech, Cat#RK001003). These cells were cultivated in a medium supplemented with puromycin (4 µg/ml) until a population of resistant cells was obtained. PC9 cells were maintained in Dulbecco's Modified Eagle Medium (DMEM), whereas HCC827 cells were cultured in RPMI-1640 medium. Both media were supplemented with 10% fetal bovine serum, 1% L-glutamine, and penicillin-streptomycin, and cells were incubated at 37 °C in a humidified incubator with 5% CO₂.

For the NSCLC cancer tissues samples in this study, resection surgeries were conducted on a cohort of patients who had not received prior treatment. All EGFR-mutant patients carried an exon 19 deletion. Tumor specimens were specifically procured from primary tumors and classified in accordance with criteria delineated by the World Health Organization. Hematoxylin and eosin (H&E) staining and immunohistochemistry (IHC) staining were performed on partially paraffin-embedded samples from 12 patients. The remaining samples were snap-frozen in liquid nitrogen for subsequent western blot analysis.

To characterize protein expression changes associated with acquired resistance to EGFR-TKIs, formalin-fixed, paraffin-embedded (FFPE) tumor specimens were collected from six patients with histologically confirmed lung adenocarcinoma (LUAD). For each patient, paired tumor samples were obtained at two clinically defined time points: prior to initiation of EGFR-TKI therapy and following the onset of drug resistance, as confirmed by radiological and pathological criteria. Immunohistochemical (IHC) staining was performed to evaluate protein expression. Additionally, for one patient, matched tumor specimens were snap-frozen in liquid nitrogen immediately upon surgical resection for subsequent western blot analysis.

Ethical approval for this study was acquired from the local research ethics committee (Approval number: 83230342), with written informed consent obtained from all patients in adherence to the principles outlined in the Declaration of Helsinki.

### Hematoxylin and Eosin (H&E) staining and Immunohistochemistry (IHC) staining

Tissue samples embedded in paraffin were meticulously sectioned to 3 µm thickness to facilitate subsequent experimental procedures. The visualization of tissue morphology was achieved using an automated Autostainer (Leica, Cat#CV5030) for Hematoxylin and eosin (HE) staining, with Hematoxylin employed to highlight cell nuclei and eosin utilized for cytoplasmic staining.

For the immunohistochemistry (IHC) experiments, the tissue sections underwent a series of meticulous steps. This involved oven-drying and dewaxing with xylene, followed by rehydration utilizing a gradient of anhydrous ethanol. Antigen retrieval was executed by boiling the sections in a sodium citrate repair solution for 2 minutes, and then the sections were rinsed with H_2_O until reaching room temperature. The sections were further treated by blocking with 0.3% H_2_O_2_ for 10 minutes, followed by incubation with specific primary antibodies, including anti-LAPTM4B antibody (Atlas Antibodies, Cat#AMAb91356, 1:400), anti-ATP1A1 antibody (Proteintech Cat#55187-1-AP, 1:200), and anti-Ki67 antibody (Servicebio Cat#GB111499, 1:500), for 2.5 hours at room temperature. After thorough washing with PBS, a secondary HRP-conjugated antibody (MXB, Cat#KIT-5010) was applied and incubated for 30 minutes at room temperature. Subsequently, staining was performed for 2 minutes using the DAB development kit (Proteintech, Cat#PR30010) and counterstained with hematoxylin for 10 seconds. The sections were dehydrated using a gradient of anhydrous ethanol and sealed with neutral resin gel.

For the analysis of the IHC results, ten random fields of view per sample were captured utilizing a microscopy system (Leica, Cat#DM2500) and quantified using the "IHC Profiler" plugin in ImageJ software version 1.53C (NIH, Bethesda, MD; http://imagej.nih.gov/ij). The IHC signal was quantified by normalizing it to the total cell number per image. To ensure accurate interpretation, all H&E and IHC images were independently evaluated by two pathologists.

### Q-PCR

Total RNA was extracted from cells or tissues using RNA isolater Total RNA Extraction Reagent (Vazyme, Cat#R401-01). cDNA synthesis was carried out with Hifair®V one-step RT-gDNA digestion SuperMix for QPCR (Yeasen, Cat#11141ES60), followed by real-time PCR using the StepOne Real-Time PCR System (Applied Biosystems, Foster City, CA, USA). Primer sequences utilized in this study were listed in** Supplementary Table S1**.

### Transmission electron microscope measurement

Transmission electron microscopy (TEM), conducted by HITACHI, was utilized to explore the intricacies of lysosomal ultrastructure. Initially, cells underwent PBS washing and subsequent centrifugation to form a pellet, which was then reconstituted in TEM fixative and preserved at 4°C. The application of agarose pre-embedding involved additional processing steps: the fixed cells were centrifuged, the supernatant discarded, and the pellet washed in a 0.1 M phosphate buffer (PB) with a pH of 7.4, with this rinsing sequence repeated thrice. A 1% agarose solution was meticulously prepared, added to the EP tube, and the resultant agarose carefully enveloped the suspended pellet before solidification. Post-fixation with 1% OsO4 in 0.1 M PB (pH 7.4) under light-shielded conditions occurred for 2 hours at room temperature. Subsequent to OsO4 removal, a triple rinse in 0.1 M PB (pH 7.4) for 15 minutes each was performed. Following dehydration, resin penetration, and embedding at ambient temperature, polymerization was executed to ensure sample stabilization. Ultra-thin sections were then procured, subjected to staining techniques, and visualized via TEM utilizing cuprum grids. Finally, lysosomal diameter and ultrastructural features were meticulously assessed and quantified using ImageJ software.

### The evaluation of lysosomal pH

The lysosomal pH was assessed utilizing the lysosome pH detection assay kit (Bestbio, Cat#BB-481215) according to the manufacturer's instructions. In brief, the pH probe was diluted in Hank's Balanced Salt Solution (HBSS) at 1:1000, cells were collected and incubated with diluted pH probe at 37°C for 5 minutes, followed by flow cytometry analysis. The ratio of fluorescein emission under excitation at 525 nm and 450 nm (F525/F450) was calculated to determine the lysosomal pH levels. The F525/F450 ratio exhibited an inverse relationship with pH values, as described in previous study 37.

### The high-throughput drug screen experiment

The drug screening experiment were conducted by our in-house system from the University of Helsinki, as described previously 38,39. Cells were seeded in 384-well plates using a robotic system and incubated with an oncology collection drug library comprising 527 compounds, including FDA-approved drugs and emerging investigational agents, with five different concentrations for each individual compound. The drug administration was performed by a sonic robotic system, to ensure the high-content and high-efficiency screening procedure. After the drug treatment, the cells were imaged by the PerkinElmer Opera Phenix high-content microscopy system. To ensure a high-efficiency and unbiased data collection procedure, we employed automated analysis using "Cell profiler" and "Python Script" to quantify the intensity of sfGFP and mCherry in each individual cell.

In this drug screening experiment, the specificity of fluorescence as well as the imaging and data analysis methods were validated by siRNAs against LAPTM4B or CD63.

### *In vivo* xenograft experiment

All xenograft tumor growth experiments adhered to the guidelines for animal experimentation and were ethically approved by the Anhui Medical University Ethics Committee (Approval number: LISC20231014).

Twenty female BALB/c nude mice (aged 5 weeks) were procured from GemPharmatech Company (Nanjing, China) and maintained under standard housing conditions. Subsequently, the mice were subcutaneously injected with 1x10^7^ PC9-GR cells. Following an 11-day interval, palpable tumors had developed, prompting the random allocation of the mice into four distinct subgroups. These subgroups were respectively treated with gefitinib (12.5mg/kg), copanlisib (6mg/kg), their combination (Gefitinib+Copanlisib), or served as the control group.

The administration of gefitinib was carried out via intragastric route, while copanlisib was administered intraperitoneally; the control group were administered using the same method as the corresponding treatment group. The therapeutic regimens were sustained for a period of 10 days until the mice were humanely euthanized. It is noteworthy that two mice succumbed during the treatment phase, and variations in tumor development were observed among the different experimental groups. We thereafter selected the eight most substantial tumors for further analysis.

### Statistical analysis

All the data are presented as the mean ± standard error of the mean (SEM) from at least three independent experiments. Statistical significance was calculated using the Student's t-test for pairwise comparisons. * indicates *p* < 0.05, ** indicates *p* < 0.01, *** indicates *p* < 0.001.

Additional detailed descriptions of methods are in **Supplementary Materials and Methods**.

## Results

### The lysosomal protein LAPTM4B is upregulated in EGFR-TKI resistant NSCLC patients

**Figure 1:**
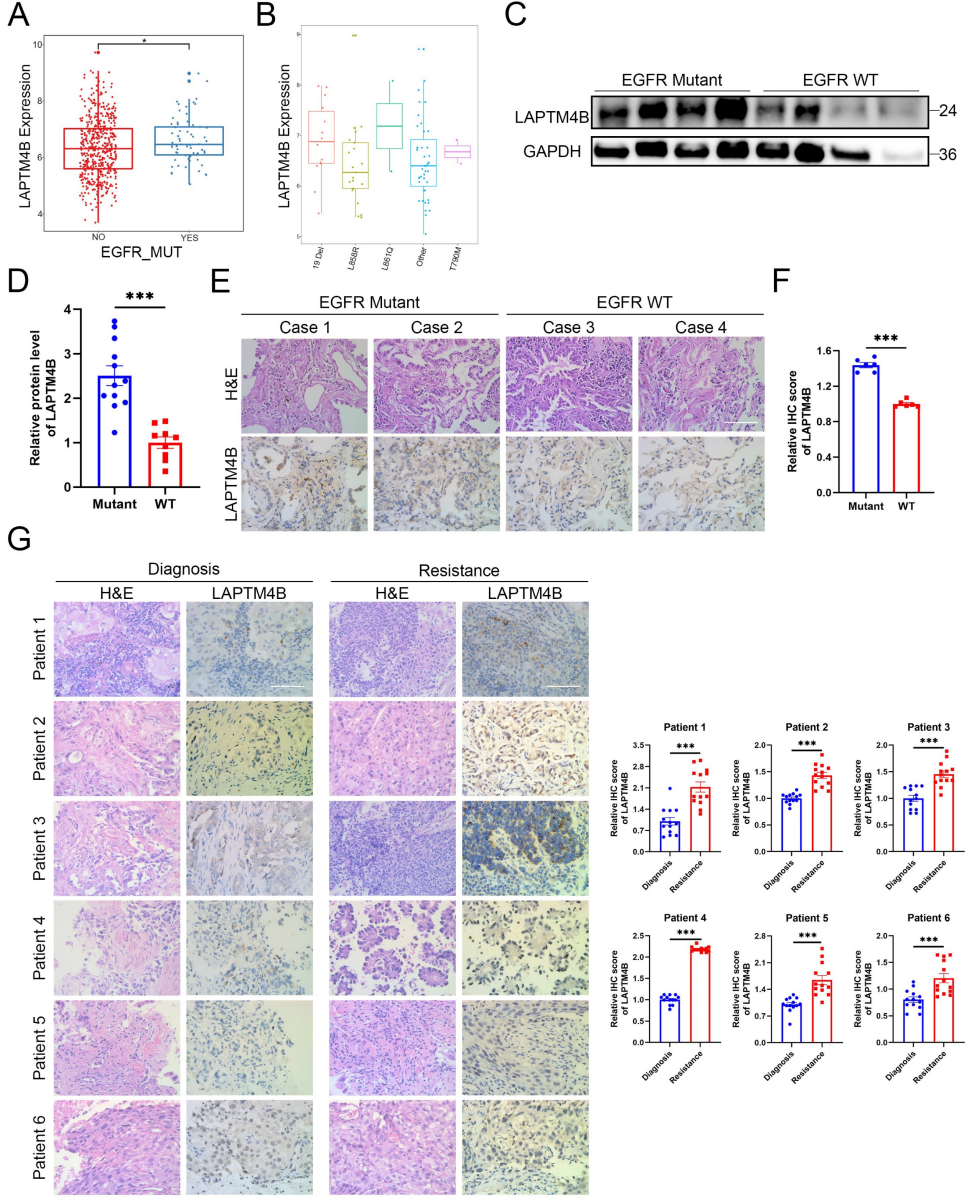
**The lysosomal protein LAPTM4B is upregulated in EGFR-TKI resistant NSCLC patients. (A)**LAPTM4B expression in EGFR mutant and EGFR wild-type (WT) NSCLC patients. Unit: Log_2_(FPKM+1). **(B)**LAPTM4B expression in NSCLC patients with different types of EGFR mutation. Unit: Log_2_(norm_count+1). **(C)**Western blot analysis of LAPTM4B levels in EGFR mutant and EGFR WT NSCLC tumors. The EGFR mutation type in the mutant cohort is exon 19 deletion (19 Del). **(D)**Quantification of **(C)**. Note: The experiment was technically repeated in triplicate, and data from all replicates were pooled for analysis. One “EGFR WT” sample with extremely low LAPTM4B and GAPDH signals was excluded from statistical analysis. **(E)**Immunohistochemistry (IHC) staining of LAPTM4B in tumor tissue of EGFR mutant and EGFR WT patients. Scale bar: 100 µm. Mutation type is exon 19 deletion (19 Del). **(F)**Quantification of LAPTM4B expression in IHC results from **(E)**. **(G)**Immunohistochemical analysis of LAPTM4B expression in matched tumor biopsies collected from six EGFR-mutant NSCLC patients at initial diagnosis and after the development of clinical resistance to EGFR-TKI therapy. Scale bar: 100 µm. Quantification of n=3 experiments, at least 13 images were analyzed per samples. Mean ± SEM.

To explore the potential involvement of LAPTM4B in EGFR-TKI resistance, we first analyzed its expression relative to EGFR mutation status using The Cancer Genome Atlas (TCGA). This analysis revealed a significant overexpression of LAPTM4B in EGFR-mutant NSCLC tumors (**Fig. 1A**), most prominently in cases harboring L861Q mutation and exon 19 deletions (**Fig. 1B**). Notably, L861Q occurs in only approximately 1-3% of EGFR-mutant NSCLCs 40, whereas exon 19 deletions are far more prevalent (about 50%) 41, representing the primary activating mutations.

Given the rarity of L861Q, we prioritized exon 19 deletions in our subsequent functional and mechanistic studies. Analysis of clinical NSCLC tumor specimens confirmed elevated LAPTM4B protein expression in EGFR-mutant (exon 19 deletion) samples, relative to EGFR wild-type samples. These findings were validated by both immunoblotting (**Fig. 1C-D**) and immunohistochemistry (**Fig. 1E-F**).

To assess the dynamic regulation of LAPTM4B during the acquisition of drug resistance, we collected matched tumor tissues from six patients with histologically confirmed lung adenocarcinoma, all carrying EGFR 19Del mutations (**Supplementary Table S2**). These patients initially responded to EGFR-TKI therapy but subsequently developed disease progression after 6 to 30 months of treatment, indicative of acquired resistance (**Supplementary Fig. S1A**). Immunohistochemical analysis revealed a consistent and pronounced increase in LAPTM4B expression in post-resistance samples compared to the corresponding pre-treatment biopsies from the same individual (**Fig. 1G**). Additionally, tumor samples from one patient (Patient #1) were also snap-frozen at the time of collection, allowing for immunoblot analysis, which independently suggested the upregulation of LAPTM4B following the development of EGFR-TKI resistance (**Supplementary Fig. S1B**).

Collectively, these findings derived from integrative bioinformatics, protein-level validation in EGFR-mutant tumors, and matched resistant tumor biopsies, suggest that LAPTM4B is a clinically relevant factor in EGFR-mutant NSCLC and may contribute to the molecular underpinnings of acquired resistance to EGFR-targeted therapy.

### EGFR-TKIs upregulate LAPTM4B by suppressing WWP2-mediated proteasomal degradation in NSCLC cell lines

**Figure 2:**
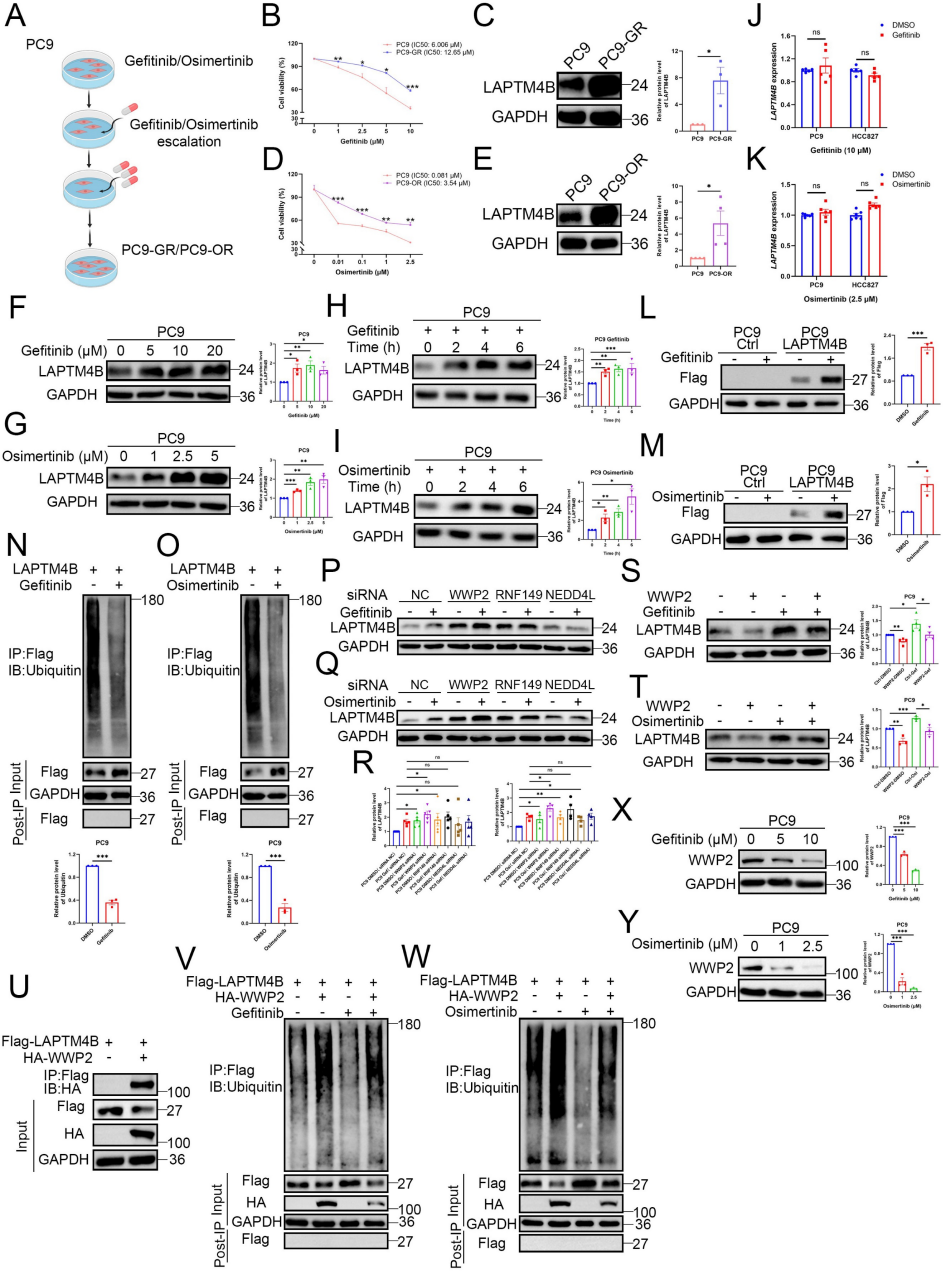
**EGFR-TKI upregulate LAPTM4B by suppressing WWP2-mediated proteasomal degradation in NSCLC cell lines. (A)**The flowchart of generating Gefitinib-resistant cells (PC9-GR) and Osimertinib-resistant cells (PC9-OR). **(B)**Cell viability in PC9 and PC9-GR incubated with gefitinib. Quantification of at least three experiments. **(C)**Western blot analysis of LAPTM4B levels in PC9-GR and the parental PC9 cells. Quantification of n=3 experiments. **(D)**Cell viability in PC9 and PC9-OR incubated with osimertinib. Quantification of at least three experiments. **(E)**Western blot analysis of LAPTM4B levels in PC9-OR and the parental PC9 cells. Quantification of n=4 experiments. **(F)**PC9 cells were treated with gefitinib for 6 h at the indicated concentration, and the expression of LAPTM4B was measured by western blotting. Quantification of n=3 experiments. **(G)**PC9 cells were treated with osimertinib for 6 h at the indicated concentration, and the expression of LAPTM4B was measured by western blotting. Quantification of n=3 experiments. **(H)**PC9 cells were treated with 10 µM gefitinib for the indicated time, and the expression of LAPTM4B was measured by western blotting. Quantification of n=3 experiments. **(I)**PC9 cells were treated with 2.5 µM osimertinib for the indicated time, and the expression of LAPTM4B was measured by western blotting. Quantification of n=3 experiments. **(J)**PC9 and HCC827 cells were treated with 10 µM gefitinib for 6 h, and LAPTM4B mRNA expression was assessed by qRT-PCR. Quantification of n=5 experiments. **(K)**PC9 and HCC827 cells were treated with 2.5 µM osimertinib for 6 h, and LAPTM4B mRNA expression was assessed by qRT-PCR. Quantification of n=6 experiments. **(L)**PC9 Ctrl and PC9 LAPTM4B cells were treated with 10 µM gefitinib for 6 h, and the expression of Flag-LAPTM4B was measured by western blotting. **(M)** PC9 Ctrl and PC9 LAPTM4B cells were treated with 2.5 µM osimertinib for 6 h, and the expression of Flag-LAPTM4B was measured by western blotting. **(N)**Flag-tagged LAPTM4B stably expressing PC9 cells were treated with 10 µM gefitinib or DMSO for 6 h. Immunoprecipitation of the cell lysate using Flag antibody, followed by immunoblotting with Ubiquitin antibody. Quantification of n=3 experiments. **(O)**Flag-tagged LAPTM4B stably expressing PC9 cells were treated with 2.5 µM osimertinib or DMSO for 6 h. Immunoprecipitation of the cell lysate using Flag antibody, followed by immunoblotting with Ubiquitin antibody. Quantification of n=3 experiments. **(P)**PC9 cells were transfected with indicated siRNA, and afterwards treated with 10 µM gefitinib or DMSO for 6 h. The expression of LAPTM4B was measured by western blotting. **(Q)**PC9 cells were transfected with indicated siRNA, and afterwards treated with 2.5 µM osimertinib or DMSO for 6 h. The expression of LAPTM4B was measured by western blotting. **(R)**Quantification of **(P)** and **(Q)**. Quantification of at least n=4 experiments. **(S)**PC9 cells were overexpressed with WWP2, treated with 10 µM gefitinib or DMSO for 6 h. The expression of LAPTM4B was measured by western blotting. Quantification of n=4 experiments. **(T)**PC9 cells were overexpressed with WWP2, treated with 2.5 µM osimertinib or DMSO for 6 h. The expression of LAPTM4B was measured by western blotting. Quantification of n=3 experiments. **(U)**Flag-tagged LAPTM4B stably expressing PC9 cells were transfected with HA-tagged WWP2. Cell lysates were immunoprecipitated with anti-Flag beads and analyzed by immunoblotting with an anti-HA antibody. **(V)**Flag-tagged LAPTM4B stably expressing PC9 cells were transfected with or without HA-tagged WWP2, and treated with 10 µM gefitinib or DMSO for 6 h. Cell lysates were immunoprecipitated with anti-Flag beads and analyzed by immunoblotting with an anti-Ubiquitin antibody. **(W)**Flag-tagged LAPTM4B stably expressing PC9 cells were transfected with or without HA-tagged WWP2, and treated with 2.5 µM osimertinib or DMSO for 6 h. Cell lysates were immunoprecipitated with anti-Flag beads and analyzed by immunoblotting with an anti-Ubiquitin antibody. **(X)**PC9 cells were treated with gefitinib for 6 h at the indicated concentration, and the expression of WWP2 was measured by western blotting. Quantification of n=3 experiments. **(Y)**PC9 cells were treated with osimertinib for 6 h at the indicated concentration, and the expression of WWP2 was measured by western blotting. Quantification of n=3 experiments.

To determine whether LAPTM4B contributes functionally to EGFR-TKIs resistance, we established gefitinib-resistant PC9 cells (PC9-GR) by gradually increasing gefitinib concentrations (**Fig. 2A**). PC9-GR cells exhibited significantly higher viability than parental PC9 cells upon gefitinib treatment (**Fig. 2B**), accompanied by a substantial upregulation of LAPTM4B expression (**Fig. 2C**). Similarly, we generated an osimertinib-resistant PC9 cell line (PC9-OR) using the same gradual drug exposure strategy (**Fig. 2A, 2D**), and observed a comparable increase in LAPTM4B protein levels (**Fig. 2E**). Importantly, LAPTM4B mRNA levels remained unchanged in both PC9-GR and PC9-OR cells compared to the parental cells (**Supplementary Fig. S2A-B**), indicating that the observed increase in protein is likely mediated by post-transcriptional mechanisms.

Prompted by these findings, we next investigated whether EGFR-TKI treatment directly regulates LAPTM4B expression in NSCLC. Both gefitinib and osimertinib induced a robust, dose- and time-dependent increase in LAPTM4B protein levels in PC9 (**Fig. 2F-I**) and HCC827 cells (**Supplementary Fig. S2C-F**). In contrast, quantitative PCR analysis revealed no significant change in LAPTM4B mRNA expression upon EGFR-TKI treatment (**Fig. 2J-K**), suggesting a post-transcriptional mechanism. Notably, EGFR-TKIs continued to elevate LAPTM4B protein levels in the presence of cycloheximide (CHX), a translation inhibitor (**Supplementary Fig. S2G-H**), further supporting a post-translational mode of regulation.

To directly evaluate protein turnover, we performed CHX-chase experiments and monitored LAPTM4B degradation over time by immunoblotting. LAPTM4B displayed a short half-life of approximately 0.5-1 hour in both PC9 and HCC827 lines (**Supplementary Fig. S2I**), consistent with the prior report in A431 cells 25. Pharmacological inhibition of protein degradation pathways revealed that both the proteasome inhibitor MG-132, and the lysosome inhibitor bafilomycin-A1, attenuated LAPTM4B degradation (**Supplementary Fig. S2J**), indicating LAPTM4B undergoes dual proteasomal and lysosomal degradation in NSCLC, in line with previous study 25.

Given the pronounced stabilization of LAPTM4B upon MG-132 treatment (**Supplementary Fig. S2J**), we hypothesized that EGFR-TKIs may regulate LAPTM4B via proteasomal pathways. To interrogate the mechanism, we generated LAPTM4B knockout (KO) monoclonal lines in PC9 and HCC827 cell backgrounds using CRISPR-Cas9n, which were validated by Sanger sequencing and immunoblotting (**Supplementary Fig. S3A-B**). We then reconstituted the KO cells with Flag-tagged LAPTM4B (**Supplementary Fig. S3C-D**). Notably, gefitinib and osimertinib significantly upregulated exogenous LAPTM4B in the stably expressing cells (**Fig. 2L-M, Supplementary Fig. S3E-F**), which is consistent with the data of endogenous LAPTM4B (**Fig. 2F-I, Supplementary Fig. S2C-F**). To determine whether LAPTM4B stabilization was linked to changes in ubiquitination, we immunoprecipitated Flag-LAPTM4B from MG-132-treated cells and assessed polyubiquitination. Both gefitinib and osimertinib markedly reduced LAPTM4B ubiquitination in PC9 (**Fig. 2N-O**) and HCC827 cells (**Supplementary Fig. S3G-H**), implicating EGFR-TKIs in suppression of proteasome-targeting modifications.

To identify the E3 ubiquitin ligase responsible for LAPTM4B degradation, we conducted immunoprecipitation (IP) of Flag-tagged LAPTM4B, followed by mass spectrometry analysis. Several candidate E3 ligases, including RNF149 and WWP2, were enriched in both this analysis (**Supplementary Table S3**) and previous LAPTM4B interactome dataset 24. Given prior reports implicating possible regulation between NEDD4L and LAPTM4B 42,43, we selected RNF149, WWP2, and NEDD4L for further siRNA screening **(Supplementary Fig. S3I)**. Notably, WWP2 knockdown significantly increased LAPTM4B protein levels under both basal and EGFR-TKI-treated conditions, whereas RNF149 and NEDD4L knockdown had minimal effects (**Fig. 2P-R**).

Conversely, ectopic expression of WWP2 attenuated the EGFR-TKI-induced accumulation of LAPTM4B protein in PC9 cells (**Fig. 2S-T**) and HCC827 cells (**Supplementary Fig. S3J-K**). Immunoprecipitation experiments further showed that LAPTM4B interacts with WWP2 (**Fig. 2U, Supplementary Fig. S3L**), supporting a functional regulatory relationship. Moreover, overexpression of WWP2 restored LAPTM4B ubiquitination levels in both PC9 (**Fig. 2V-W**) and HCC827 cells (**Supplementary Fig. S3M-N**), irrespective of EGFR-TKI treatment. Interestingly, both gefitinib and osimertinib treatment resulted in a dose-dependent reduction in WWP2 protein levels (**Fig. 2X-Y, Supplementary Fig. S3O-P**), suggesting that EGFR-TKIs stabilize LAPTM4B, at least in part, by attenuating WWP2-mediated ubiquitination and subsequent proteasomal degradation.

Collectively, these findings reveal a previously unrecognized mechanism by which EGFR-TKIs upregulate LAPTM4B protein level through attenuation of WWP2-mediated ubiquitination and proteasomal degradation, providing novel mechanistic insight into the post-translational regulation of LAPTM4B and its potential role in the development of acquired drug resistance.

### LAPTM4B promotes EGFR-TKI resistance through lysosomal acidification

**Figure 3:**
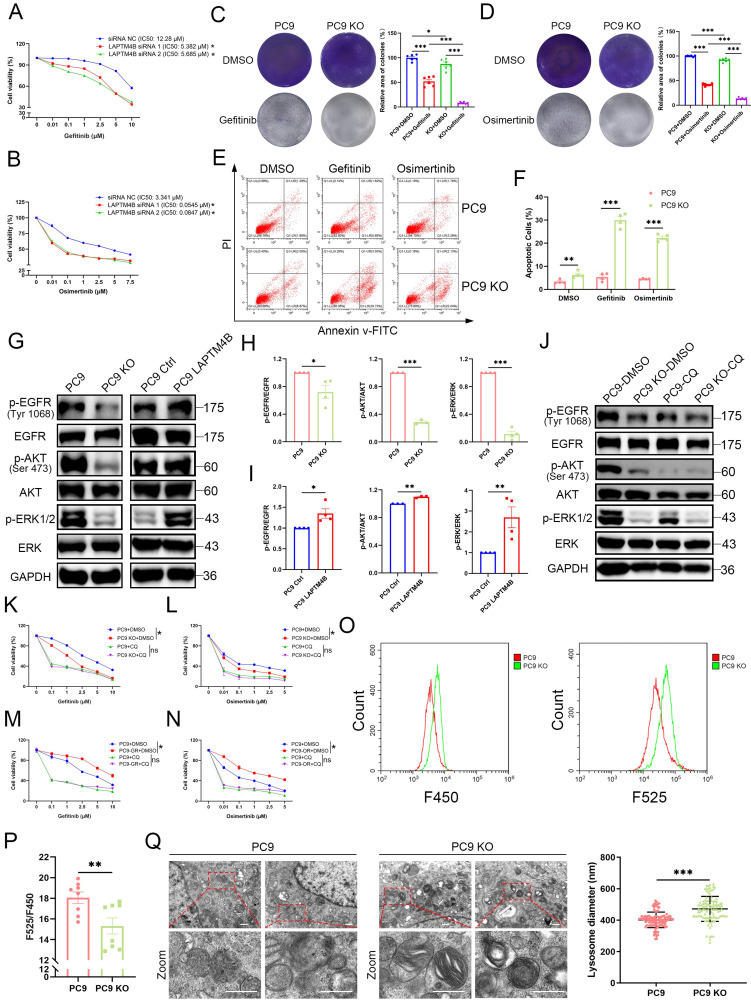
** LAPTM4B-mediated EGFR-TKI resistance depends on lysosomal acidification. (A)**Cell viability in PC9-GR transfected by siRNA NC and two independent LAPTM4B siRNAs, incubated with gefitinib. Quantification of at least three experiments. * indicates p<0.05. **(B)**Cell viability in PC9-OR transfected by siRNA NC and two independent LAPTM4B siRNAs, incubated with osimertinib. Quantification of at least three experiments. * indicates p<0.05. **(C)**Colony formation assay in WT and LAPTM4B KO PC9 cells. Cells were continuously treated with 10 µM gefitinib or DMSO for 10 days. N=6. **(D)**Colony formation assay in WT and LAPTM4B KO PC9 cells. Cells were continuously treated with 1 µM osimertinib or DMSO for 10 days. N=6. **(E)**Cell apoptosis in WT and KO PC9 cells upon the treatment with gefitinib or osimertinib. **(F)**Quantification of **(E)**. N=4 experiments. **(G)**Immunoblotting of p-EGFR, p-AKT, and p-ERK in PC9 and PC9 KO cells, as well as in LAPTM4B stably expressing PC9 cells and their respective controls. Phosphorylation sites for p-EGFR and p-AKT are indicated in the figure. The phosphorylation sites for p-ERK1/2 are (Thr202/Tyr204)/ (Thr185/Tyr187). **(H)**Quantification in PC9 and PC9 KO cells. N=3. **(I)**Quantification in LAPTM4B stably expressing PC9 and the control cells. N=3. **(J)**Immunoblotting of p-EGFR, p-AKT, p-ERK in PC9 and PC9 KO cells, treated with chloroquine (CQ) or DMSO for 48h. **(K)**Cell viability of wild-type (WT) and LAPTM4B knockout (KO) PC9 cells treated with gefitinib, with or without chloroquine (CQ). Data represent the mean ± SEM from 3 independent experiments. Note: All viability values were normalized to their respective DMSO-treated controls, such that each DMSO-treated group (WT and KO) is set to 100% **(L)**Cell viability in WT and LAPTM4B KO PC9 cells incubated with osimertinib, together with or without CQ. N=3. **(M)**Cell viability measured in PC9 and PC9-GR cells incubated with gefitinib, together with or without CQ. N=3. **(N)**Cell viability in PC9 and PC9-OR cells incubated with osimertinib, together with or without CQ. N=3. **(O)**Flow cytometric analysis of the ratio of fluorescein emission when excited by 525 and 450 nm light (F525/F450). Note: The F525/F450 ratio was inversely correlated with lysosomal pH value. **(P)**The statistical analysis of F525/F450. N=8. **(Q)**Transmission electron microscopy analysis of lysosome ultrastructure in WT and KO PC9 cells. Quantification of n=4 experiments. Scale bar: 0.5 µm. The region in the dashed red box is amplified in the lower panel.

To evaluate whether LAPTM4B is required for EGFR-TKI resistance, we silenced LAPTM4B using two independent siRNAs, which were validated at both the transcript and protein levels (**Supplementary Fig. S4A-D**). Strikingly, LAPTM4B depletion re-sensitized PC9-GR cells to gefitinib (**Fig. 3A**) and PC9-OR cells to osimertinib (**Fig. 3B**).

We next measured the drug resistance in LAPTM4B KO cells. As anticipated, our experiments using Cell Counting Kit-8 (CCK8) revealed that LAPTM4B-deficient cells exhibited increased sensitivity to first-generation (gefitinib) (**Supplementary Fig. S4E-F**) and third-generation (osimertinib) EGFR-TKIs (**Supplementary Fig. S4G-H**). These findings were further supported by colony formation assays in which the cells were continuously treated with gefitinib or osimertinib for a duration of 10 days, which demonstrated a significant effect of LAPTM4B on the resistance to EGFR-TKIs (**Fig. 3C-D**). Similar results were obtained in HCC827 cells (**Supplementary Fig. S4I-J**), confirming the specificity of LAPTM4B's role in drug resistance.

To further understand the mechanism underlying heightened TKI sensitivity, we conducted flow cytometry-based apoptosis assays. Upon EGFR-TKI treatment, LAPTM4B KO cells demonstrated a significantly increased cell apoptosis rate (**Fig. 3E-F**), which is in line with elevated levels of cleaved Caspase-3/7 (**Supplementary Fig. S4K**).

Conversely, overexpression of LAPTM4B in wild-type PC9 and HCC827 cells (**Supplementary Fig. S4L-M**) conferred enhanced resistance to both gefitinib and osimertinib, as shown by dose-responsive survival curves in CCK-8 assays (**Supplementary Fig. S4N-Q**).

Given that EGFR-TKIs primarily function by inhibiting EGFR phosphorylation and its downstream signaling, we next assessed whether LAPTM4B influences these pathways. Notably, LAPTM4B depletion led to reduced phosphorylation of EGFR (p-EGFR), AKT (p-AKT), and ERK (p-ERK), whereas total EGFR, AKT, and ERK protein levels remained unchanged (**Fig. 3G-H, Supplementary Fig. S4R**). Conversely, overexpression of LAPTM4B restored p-EGFR levels and reactivated downstream signaling (**Fig. 3G, 3I**). Lysosomal acidification is a well-documented factor in drug resistance 8. To assess whether LAPTM4B-mediated resistance is dependent on lysosomal acidification, we treated WT and LAPTM4B KO PC9 cells with chloroquine (CQ), a lysosomal acidification inhibitor, and examined the impact on p-EGFR signaling. CQ treatment abolished the differences in p-EGFR levels and downstream pathway activation between WT and LAPTM4B KO PC9 cells (**Fig. 3J**). It is worth noting that basal ERK phosphorylation level was already low in LAPTM4B KO cells, with no appreciable reduction upon CQ treatment (**Fig. 3J**). Importantly, parallel experiments in HCC827 cells yielded similar results (**Supplementary Fig. S5A**). Moreover, when cells were exposed to gefitinib or osimertinib, LAPTM4B still sustained p-EGFR signaling in the presence of EGFR-TKIs treatment (**Supplementary Fig. S5B-E**). Together, these findings indicate that LAPTM4B promotes EGFR-TKI resistance by sustaining EGFR phosphorylation, a process that critically depends on intact lysosomal function.

To further investigate the role of lysosomal acidification in LAPTM4B-mediated EGFR-TKI resistance, we assessed drug resistance in the presence of both CQ and EGFR-TKIs. Strikingly, CQ treatment markedly reduced LAPTM4B-dependent resistance to gefitinib (**Fig. 3K**) and osimertinib (**Fig. 3L**). Similar results were obtained in drug-resistant cell lines, CQ administration significantly sensitized PC9-GR cells to gefitinib (**Fig. 3M**) and PC9-OR cells to osimertinib (**Fig. 3N**).

To complement our experimental findings, we analyzed single-cell RNA sequencing data from a recent study 44, which identified distinct subpopulations of NSCLC cells with enriched gene ontology (GO) terms related to "Lysosome" and "Lysosome membrane" in TKI-resistant populations (**Supplementary Fig. S5F**). Additionally, reanalysis of publicly available datasets (GSE database) indicated a strong association between TKI resistance and lysosomal pathways, particularly “Lysosome” and “Lysosome lumen” (**Supplementary Fig. S5G**). These bioinformatics analyses further support the role of lysosomes in EGFR-TKI resistance.

Finally, we directly assessed lysosomal acidification using a fluorescent pH probe and transmission electron microscopy (TEM). LAPTM4B KO cells exhibited elevated lysosomal pH (indicated by a decrease in F525/F450 fluorescence ratio) compared to WT cells (**Fig. 3O-P**). Ultrastructural analysis by TEM further revealed enlarged and swollen lysosomes accumulating in LAPTM4B-deficient cells (**Fig. 3Q, Supplementary Fig. S5H-I**).

Collectively, these results establish that LAPTM4B promotes EGFR-TKI resistance by maintaining lysosomal acidification, thereby sustaining p-EGFR signaling and supporting cancer cell survival under TKI treatment.

### LAPTM4B interacts with and recruits ATP1A1 to the lysosome

**Figure 4:**
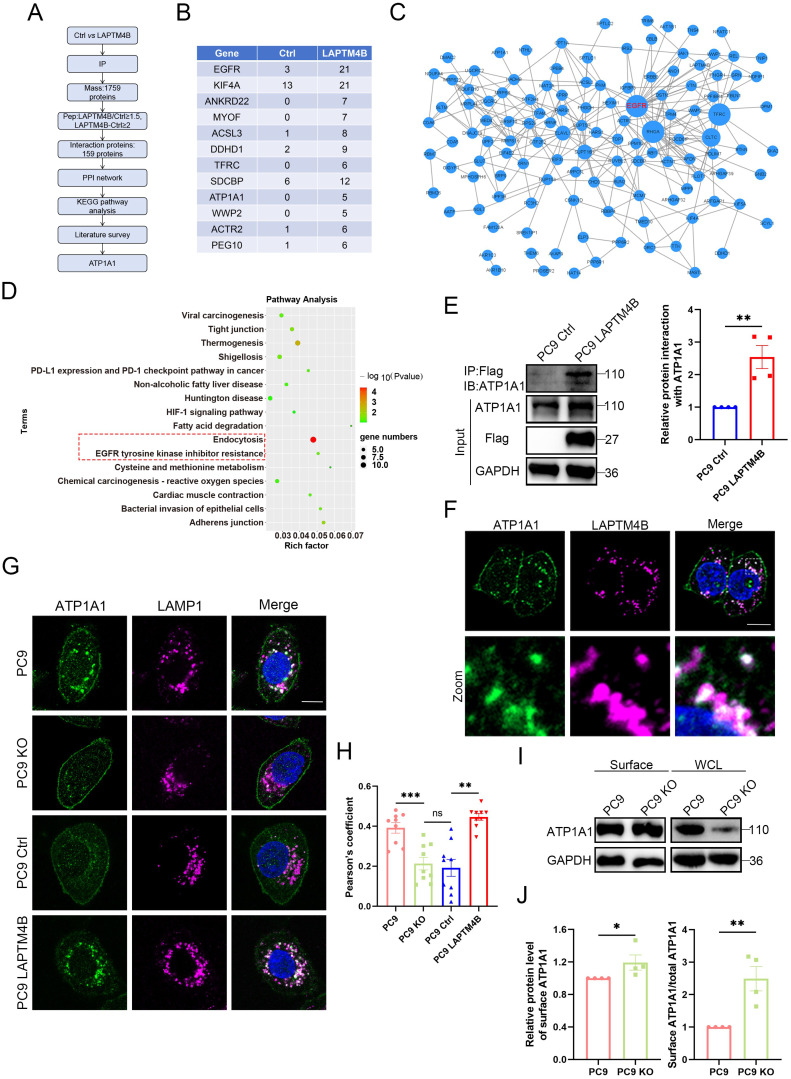
** LAPTM4B interacts with and recruits ATP1A1 to the lysosome. (A)** The flow chart of identifying interacting partners of LAPTM4B. **(B)**Top 12 candidate of potential LAPTM4B interacting proteins after applying the threshold of peptides (LAPTM4B/Ctrl>=1.5, LAPTM4B - Ctrl>=2). **(C)**The protein-protein interaction (PPI) network of potential interacting proteins. **(D)**KEGG analysis of the enriched protein. The “EGFR tyrosine kinase inhibitor resistance” and “Endocytosis” pathways were highlighted by red dashed box. **(E)**Immunoprecipitation of Flag-tagged LAPTM4B stably expressing PC9 cells and control cells using Flag antibody, followed by immunoblotting with ATP1A1 antibody. Left panel: Representative experiment. Right panel: Quantification of n=4 experiments, presented as mean ± SEM, data normalized to "Ctrl". *p*(Ctrl, LAPTM4B)=0.0024. **(F)**Immunofluorescence staining of Flag-tagged LAPTM4B stably expressing cells using anti-Flag antibody (magenta) and anti-ATP1A1 antibody (green). Scale bar: 10 µm. The region in the dashed white box is amplified in the lower panel. **(G)**Immunofluorescence staining of ATP1A1 and LAMP1 in WT and LAPTM4B KO PC9 cells, as well in LAPTM4B stably expressing cells and the control. Scale bar: 10 µm. **(H)**Pearson's coefficient of ATP1A1 and LAMP1. Quantification of n=4 experiments, mean ± SEM, at least 36 cells per group were analyzed. *p*(PC9, PC9 KO)=0.0002. *p*(Ctrl, LAPTM4B)=2.13E-05. **(I)**The plasma-membrane isolation experiment was performed on WT and LAPTM4B KO PC9 cells, the surface and total ATP1A1 levels were analyzed by western blot. **(J)**Quantification of cell-surface ATP1A1 levels, and the relative amounts of cell-surface ATP1A1 (normalized to total protein) were performed in WT and LAPTM4B KO cells; mean ± SEM, n = 4. For cell-surface ATP1A1, *p*(PC9, PC9 KO)=0.0422; For relative amounts of cell-surface ATP1A1, *p*(PC9, PC9 KO)=0.0002.

To elucidate the molecular mechanism by which LAPTM4B modulates lysosomal acidification and EGFR-TKI resistance, we hypothesized that LAPTM4B may interact with key ion transporters or proteins controlling lysosomal pH. We further analyzed the data from immunoprecipitation - mass spectrometry assay (**Supplementary Table S3**). A protein was considered a potential LAPTM4B interactor if its peptide count in LAPTM4B-expressing cells was at least 1.5-fold higher than in the control group, with a minimum absolute difference of two peptides. Using this threshold, we identified 159 candidate LAPTM4B-interacting proteins (**Fig. 4A, Supplementary Table S3**), with the top 12 enriched candidates displayed in **Fig. 4B**. Notably, among the identified proteins, several identified proteins were ion transporters or channels implicated in electrochemical gradient maintenance and lysosomal function, including ATP1A1 (the α1 subunit of Na⁺/K⁺-ATPase) and Myoferlin (MYOF) (**Fig. 4B**), suggesting that LAPTM4B may directly engage ion transporters to regulate lysosomal homeostasis.

To further characterize the functional interactions among these candidate proteins, we constructed a protein-protein interaction (PPI) network using the STRING database and visualized the interactions using Cytoscape 3.9.0. Interestingly, EGFR was identified as the central hub within this network (**Fig. 4C**). Subsequent KEGG pathway enrichment analysis revealed that “Endocytosis” and “EGFR tyrosine kinase inhibitor resistance” were among the top enriched pathways (**Fig. 4D**, highlighted in red dashed lines). Given that many of the identified proteins have known roles in EGFR-TKI resistance, we conducted a literature-based filtering process, ultimately identifying ATP1A1 as a key candidate for further investigation.

To validate the protein interaction between LAPTM4B and ATP1A1, we first performed co-immunoprecipitation (co-IP) using LAPTM4B as bait, which showed protein interaction with ATP1A1 (**Fig. 4E**). We then conducted a reciprocal co-IP—immunoprecipitating ATP1A1 and probing for LAPTM4B—which provided further verification of their interaction (**Supplementary Fig. S6A**). Consistent with these findings, immunofluorescence-based co-localization analysis revealed substantial spatial overlap between LAPTM4B and ATP1A1 within the lysosomal compartment (**Fig. 4F**).

To assess the functional consequence, we examined the subcellular localization of ATP1A1 in LAPTM4B WT and KO cells. Strikingly, LAPTM4B KO cells exhibited reduced intracellular ATP1A1 levels, whereas overexpression of LAPTM4B enhanced ATP1A1 internalization (**Fig. 4G**). Moreover, Pearson's correlation coefficient analysis between ATP1A1 and LAMP1 (a lysosomal marker) revealed a significant decrease in ATP1A1-lysosome co-localization in LAPTM4B KO cells, an effect that was rescued upon re-expression of LAPTM4B (**Fig. 4H**).

To further quantify ATP1A1 distribution, we utilized plasma membrane isolation assays to measure cell surface ATP1A1 levels. Notably, the proportion of ATP1A1 localized to the plasma membrane (relative to total ATP1A1) was significantly elevated in LAPTM4B KO cells (**Fig. 4I-J**). These findings suggest that LAPTM4B facilitates ATP1A1 internalization, leading to its recruitment to lysosomes.

Collectively, these findings establish that LAPTM4B directly interacts with ATP1A1 and regulates its intracellular trafficking, promoting lysosomal accumulation. This mechanism provides a novel regulatory axis by which LAPTM4B modulates lysosomal acidification and contributes to EGFR-TKI resistance in NSCLC.

### LAPTM4B stabilizes ATP1A1 protein via suppressing TRIM8-mediated K63-linked ubiquitination and proteasomal degradation

**Figure 5:**
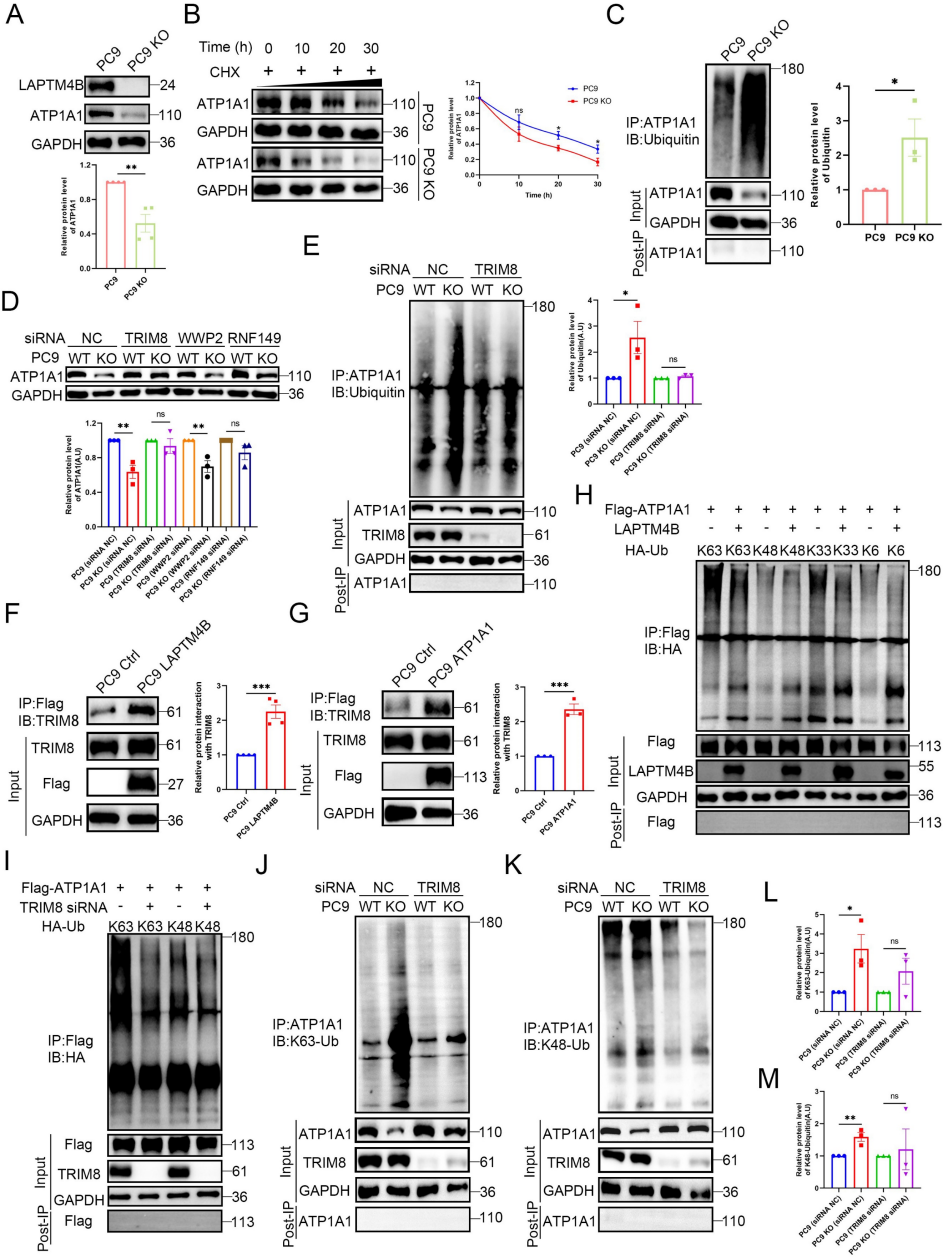
**LAPTM4B stabilizes ATP1A1 protein via suppressing TRIM8-mediated K63-linked ubiquitination and proteasomal degradation. (A)**Western blotting analysis of ATP1A1 levels in LAPTM4B-depleted PC9 cells. Quantification of n=4 experiments. **(B)**Western blot analysis of ATP1A1 levels in WT and LAPTM4B KO PC9 cells treated with cycloheximide (CHX). N=3. **(C)**Immunoprecipitation of WT and LAPTM4B KO PC9 cells using anti-ATP1A1 antibody, followed by immunoblotting with Ubiquitin antibody. N=3. **(D)**WT and LAPTM4B KO PC9 cells were transfected with indicated siRNA, and ATP1A1 levels were measured by immunoblotting. N=3. *Note*: “WT” denotes wild-type cells, “KO” denotes LAPTM4B knockout cells, and “TRIM8”, “WWP2”, “RNF149”, and “NC” refer to the siRNAs used in transfection. NC: Negative Control. **(E)**WT and LAPTM4B KO PC9 cells were transfected with indicated siRNA. Immunoprecipitation of the cell lysate using ATP1A1 antibody, followed by immunoblotting with Ubiquitin antibody. N=3. **(F)**Immunoprecipitation of Flag-tagged LAPTM4B stably expressing PC9 cells and control cells using anti-Flag beads, followed by immunoblotting with TRIM8 antibody. N=4. **(G)**Immunoprecipitation of Flag-tagged ATP1A1 stably expressing PC9 cells and control cells using anti-Flag beads, followed by immunoblotting with TRIM8 antibody. N=3. **(H)**293T cells were transfected with ATP1A1-Flag, LAPTM4B, together with HA tagged ubiquitin mutants expressing a single lysine residue, cell lysates were analyzed by immunoprecipitation by anti-Flag beads, and followed by immunoblotting with HA antibody. **(I)**293T cells were transfected with ATP1A1-Flag with HA tagged ubiquitin mutants expressing a single lysine residue, together with TRIM8 siRNA, cell lysates were analyzed by immunoprecipitation by anti-Flag beads, and followed by western blot with HA antibody. **(J)**WT and LAPTM4B KO PC9 cells were transfected with indicated siRNA. Immunoprecipitation of the cell lysate using ATP1A1 antibody, followed by immunoblotting with K63-linkage specific polyubiquitin antibody. **(K)**WT and LAPTM4B KO PC9 cells were transfected with indicated siRNA. Immunoprecipitation of the cell lysate using ATP1A1 antibody, followed by immunoblotting with K48-linkage specific polyubiquitin antibody. **(L)**Quantification of K63-polyubiquitin from at least three experiments. * indicates p<0.05. **(M)**Quantification of K48-polyubiquitin from at least three experiments. ** indicates p<0.01.

We then examined whether LAPTM4B regulates ATP1A1 protein levels. Depletion of LAPTM4B significantly reduced ATP1A1 in both PC9 and HCC827 cells (**Fig. 5A**, **Supplementary Fig. S7A**), whereas overexpression of LAPTM4B increased ATP1A1 levels (**Supplementary Fig. S7B**). Q-PCR experiments reported no significant difference of ATP1A1 transcript level in LAPTM4B depleted or overexpressing cells (**Supplementary Fig. S7C-D**). These findings suggested that LAPTM4B regulates ATP1A1 protein stability and degradation.

To assess the protein half-life, we performed a cycloheximide (CHX) chase assay and found that ATP1A1 has a half-life of approximately 18 hours in PC9 cells (**Supplementary Fig. S7E**), consistent with a previous study in U87MG cells 45. To further identify the pathway of ATP1A1 protein degradation, we treated cells with MG-132 (a proteasomal inhibitor, 20 μM) or Bafilomycin A1 (a lysosomal inhibitor, 1 μM) in combination with CHX. Our results showed that MG-132 treatment, but not Bafilomycin A1 treatment, significantly decelerated the ATP1A1 degradation (**Supplementary Fig. S7F**), indicating that ATP1A1 is degraded through the ubiquitin-proteasomal system.

Next, we investigated whether the proteasomal degradation pathway of ATP1A1 is regulated by LAPTM4B. Intriguingly, LAPTM4B depletion enhanced ATP1A1 degradation in cycloheximide-treated cells, suggesting that LAPTM4B stabilize ATP1A1 protein by antagonizing proteasomal degradation (**Fig. 5B, Supplementary Fig. S7G**). We next measured the polyubiquitination level of ATP1A1, as it is an essential step during proteasomal degradation 46. To this end, cells were treated with MG-132 to enhance polyubiquitination, and endogenous ATP1A1 was enriched through immunoprecipitation, followed by western blotting to measure ubiquitinated protein. We observed enhanced levels of ubiquitinated ATP1A1 in LAPTM4B-depleted PC9 cells (**Fig. 5C**) and HCC827 cells (**Supplementary Fig. S7H**), indicating that LAPTM4B may suppress the polyubiquitination of ATP1A1 in NSCLC cells. Interestingly, several ubiquitin E3 ligases, *e.g.* WWP2, TRIM8, and RNF149, are identified by the mass spectrometry assay as potential LAPTM4B interacting proteins (**Supplementary Table S3**), we thereafter hypothesized that certain E3 ligase is required for LAPTM4B regulated ATP1A1 protein stability. We next utilized siRNA to silence the gene expression (**Supplementary Fig. S8A, Supplementary Fig. S3I**), and found knockdown of TRIM8, but not WWP2 or RNF149, abolished the LAPTM4B-mediated difference of ATP1A1 levels in both PC9 (**Fig. 5D, Supplementary Fig. S8B**) and HCC827 cells (**Supplementary Fig. S8C-D**). To exclude off-target effects, we further employed second independent siRNA targeting TRIM8, WWP2, or RNF149 (**Supplementary Fig. S8E**), and obtained consistent results in both PC9 cells (**Supplementary Fig. S8F-G**) and HCC827 cells (**Supplementary Fig. S8H-I**). Collectively, these data indicate that LAPTM4B stabilizes ATP1A1 by antagonizing TRIM8-mediated ubiquitination and proteasomal degradation.

Importantly, the LAPTM4B dependent ATP1A1 ubiquitination could be abolished by TRIM8 silencing (**Fig. 5E**). Furthermore, immunoprecipitation experiments showed that TRIM8 is capable of interacting with LAPTM4B (**Fig. 5F**). Additionally, we generated ATP1A1 stably expressing cells using a lentivirus system (**Supplementary Fig. S8J**), and our immunoprecipitation experiments showed TRIM8 interacts with ATP1A1 (**Fig. 5G**). Moreover, we performed reciprocal co-IPs using anti-TRIM8 antibody, and our results further validated the interaction between TRIM8 and ATP1A1, LAPTM4B (**Supplementary Fig. S8K-L**).

We next set to determine the types of polyubiquitination of ATP1A1 mediated by TRIM8. To this end, we initially surveyed the literature and found TRIM8 can undergo K63-, K48-, K33- and K6- linked ubiquitination 47. Through the co-transfection of ATP1A1 with ubiquitin mutants expressing a single lysine residue, our data showed that ATP1A1 predominantly undergoes K63-linked polyubiquitination after overexpression of LAPTM4B (**Fig. 5H**), as well as after knockdown of TRIM8 (**Fig. 5I**). K48- and K63- are the major types of polyubiquitination responsible for proteasomal degradation 48, we next utilized K63-linkage or K48-linkage specific polyubiquitin antibody to detect the endogenous level of polyubiquitination. Our experiments found LAPTM4B KO cells display elevated levels of K63-linked polyubiquitination of ATP1A1, a process that can be mitigated through silencing of TRIM8 (**Fig. 5J**). Conversely, no such effects were noted in the K48-linked polyubiquitination of ATP1A1 (**Fig. 5K**). Parallel findings were observed in both PC9 (**Fig. 5J-M**) and HCC827 cells (**Supplementary Fig. S8M-N**). These data collectively demonstrate that LAPTM4B stabilizes ATP1A1 by suppressing TRIM8-mediated K63-linked ubiquitination and subsequent proteasomal degradation. It is noteworthy that the precise molecular motifs responsible for this interaction—either on ATP1A1, LAPTM4B, or TRIM8—remain undefined, and merit further structural and mutational analysis. The crosstalk between the lysosome-autophagy system and the ubiquitin-proteasome system (UPS) has been increasingly appreciated as essential for maintaining cellular proteostasis under stress, especially in cancer cells 49.

Interestingly, in a recent publication, we reported a similar mechanism in which LAPTM4B suppresses proteasomal degradation of SLC7A11, thereby counteracting ferroptosis in NSCLC 23. Together, these findings point to a broader role for LAPTM4B in bridging lysosomal function and proteasomal regulation.

### LAPTM4B-mediated EGFR-TKI resistance depends on ATP1A1

**Figure 6:**
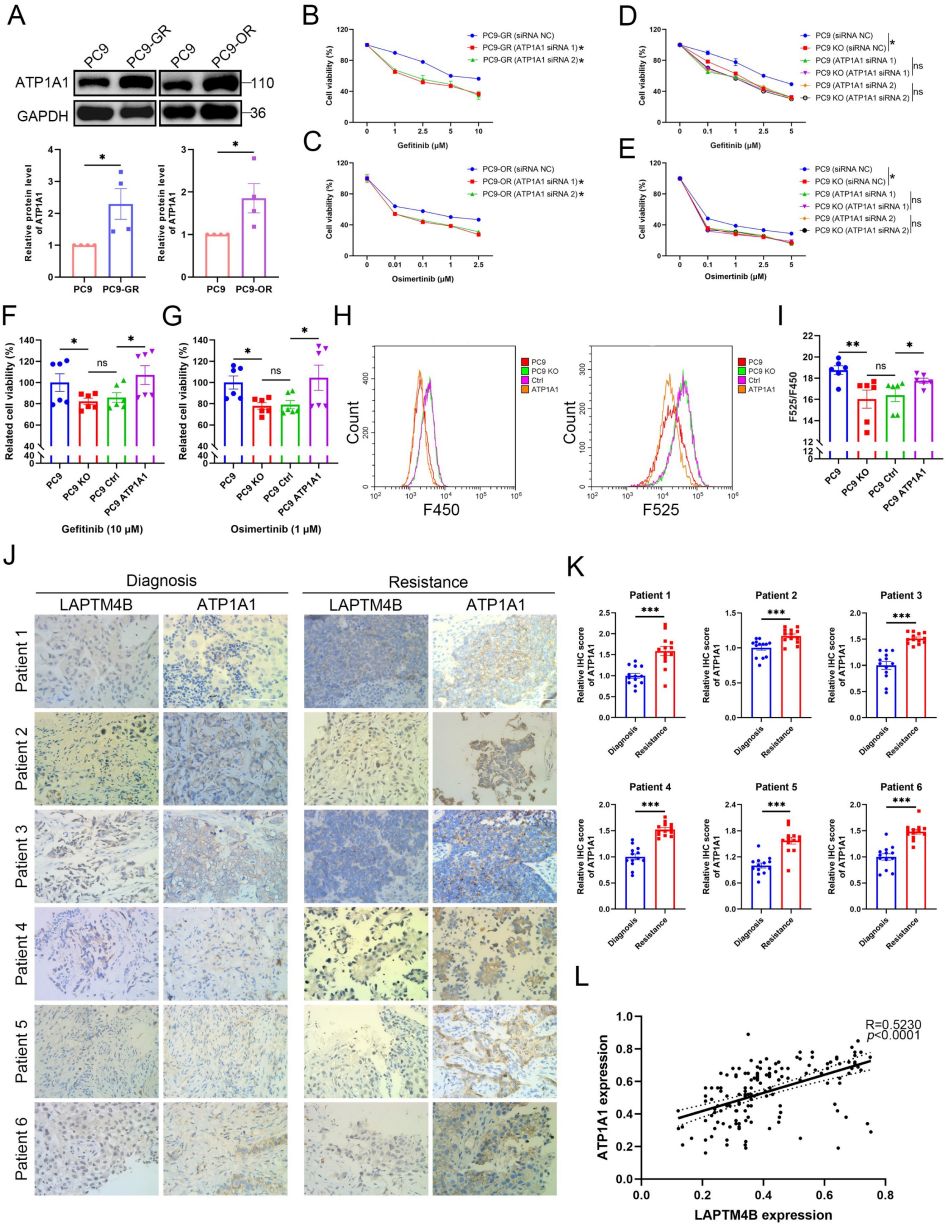
** LAPTM4B promotion of EGFR-TKIs resistance is dependent on ATP1A1. (A)**Western blot analysis of ATP1A1 levels in the resistance cells and the parental PC9 cells. Quantification of n=4 experiments. **(B)**Cell viability in PC9-GR cells transfected with siRNA NC or ATP1A1 siRNAs, incubated with gefitinib at the indicated concentrations. N = 3. **(C)**Cell viability in PC9-OR cells transfected with siRNA NC or ATP1A1 siRNAs, incubated with osimertinib at the indicated concentrations. N = 3. **(D)**Cell viability in WT or LAPTM4B KO PC9 cells transfected with siRNA NC or ATP1A1 siRNAs, treated with gefitinib at the indicated concentrations. N = 3. **(E)**Cell viability in WT or LAPTM4B KO PC9 cells transfected with siRNA NC or ATP1A1 siRNAs, treated with osimertinib at the indicated concentrations. N = 3. **(F)**Cell viability in ATP1A1 stably expressing cells (from the LAPTM4B KO background) and the control cells incubated with gefitinib. N=6. **(G)**Cell viability in ATP1A1 stably expressing cells and the control cells incubated with osimertinib. N=6. **(H)**Lysosomal pH in the indicated cells were measure by flow cytometry. **(I)**Quantification of F525/F450 in **(H)**. N=6. **(J)**Immunohistochemical analysis of LAPTM4B and ATP1A1 expression in matched tumor biopsies collected from six EGFR-mutant NSCLC patients at initial diagnosis and after the development of clinical resistance to EGFR-TKI therapy. Scale bar: 100 µm. **(K)**Quantification of ATP1A1 level from **(J)**. At least 10 images were analyzed per samples. Mean ± SEM. **(L)**Correlation between expression levels of LAPTM4B and ATP1A1 in the NSCLC patients' tissue samples from **(J)** (R=0.523, *p*<0.0001).

To investigate the role of ATP1A1 in LAPTM4B-mediated EGFR-TKIs resistance, we first measured ATP1A1 levels in the resistant cells. Both PC9-GR and PC9-OR cells exhibited elevated ATP1A1 levels relative to parental cells (**Fig. 6A**). Silencing ATP1A1 with two independent siRNAs (**Supplementary Fig. S9A-B**) resensitized PC9-GR and PC9-OR cells to gefitinib and osimertinib (**Fig. 6B-C**), demonstrating that ATP1A1 contributes to EGFR-TKI resistance.

Importantly, ATP1A1 depletion in LAPTM4B KO cells did not further increase drug sensitivity (**Fig. 6D-E, Supplementary Fig. S9C-D**), indicating that LAPTM4B and ATP1A1 function in the same pathway. Conversely, ATP1A1 overexpression in LAPTM4B KO cells (**Supplementary Fig. S8J**) restored resistance to both gefitinib and osimertinib (**Fig. 6F-G**). Accordingly, lysosomal acidification is enhanced after re-introduction of ATP1A1 in LAPTM4B KO cells (**Fig. 6H-I**). These rescue experiments establish that LAPTM4B-mediated EGFR-TKIs resistance and lysosomal acidification are dependent on ATP1A1.

To explore the clinical relevance, we assessed LAPTM4B and ATP1A1 expression in 12 paired NSCLC and adjacent normal tissues via immunohistochemistry (IHC). Representative images depicting different expression levels of LAPTM4B and ATP1A1 are presented (**Supplementary Fig. S9E**). Notably, high LAPTM4B protein expression was observed in 66.7% (8/12) of NSCLC tissues, whereas 58.3% (7/12) of adjacent normal tissues displayed low LAPTM4B expression (**Supplementary Fig. S9F**). Similarly, high ATP1A1 protein expression was observed in 75% (9/12) of NSCLC tissues, while 66.7% (8/12) of adjacent normal tissues exhibited low ATP1A1 expression (**Supplementary Fig. S9F**). Interestingly, the levels of LAPTM4B and ATP1A1 are significantly correlated (**Supplementary Fig. S9G**). These expression patterns and correlations were further corroborated by western blotting of snap-frozen patient samples (**Supplementary Fig. S9H-J**).

To investigate the clinical association of LAPTM4B and ATP1A1 with acquired EGFR-TKI resistance, we further analyzed paired tumor biopsies from six patients with EGFR-mutant NSCLC collected before treatment and after acquisition of EGFR-TKI resistance. Immunohistochemical analysis revealed marked upregulation of both LAPTM4B (**Fig. 6J, Fig. 1G**) and ATP1A1 (**Fig. 6J-K**) in post-resistance tumor samples compared to matched pre-treatment samples from the same patients. Notably, LAPTM4B and ATP1A1 expression levels were positively correlated across tumor specimens (**Fig. 6L**), further supporting a cooperative role for these two proteins.

Collectively, these data indicate that LAPTM4B and ATP1A1 are frequently co-upregulated in NSCLC and further induced upon EGFR-TKI resistance. Their coordinated expression suggests a functional interplay in maintaining lysosomal homeostasis and promoting therapeutic escape.

### An unbiased high-content screen identifies suppressors of LAPTM4B expression

Given the established oncogenic functions of LAPTM4B in multiple cancers 15,21 and its newly identified role in promoting EGFR-TKI resistance, we hypothesized that pharmacologic suppression of LAPTM4B could enhance the efficacy of EGFR-targeted therapies. To date, no compounds selectively targeting LAPTM4B have been reported. To address this gap, we aimed to identify small molecules capable of specifically downregulating LAPTM4B expression.

**Figure 7:**
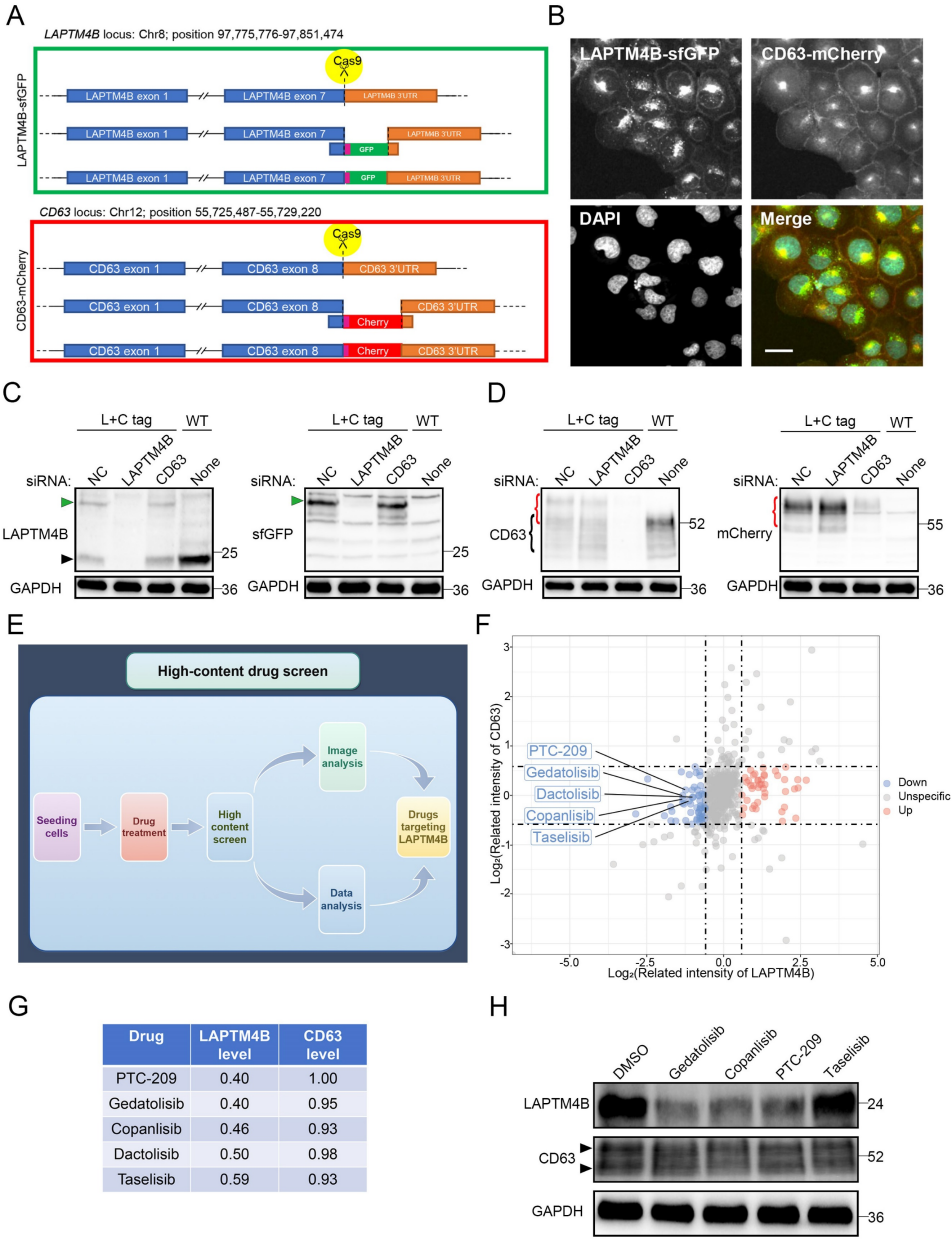
** An unbiased high-content drug screen approach to discover suppressors of LAPTM4B expression. (A)**Diagram of endogenously labeling of LAPTM4B-sfGFP and CD63-mCherry by CRISPR-Cas9. The fluorescent tags sfGFP or mCherry were knocked in between the last exon of LAPTM4B or CD63, and 3' untranslated region (3'UTR). **(B)**Confocal microscopy revealed the localization of LAPTM4B-sfGFP and control protein CD63-mCherry in endogenous labeled cells. Scale bar: 20 µm. **(C)**The endogenous labeled cell line (L+C tag) and wild-type cell line (WT) were transfected with siRNA against LAPTM4B or CD63, the protein levels were then determined by western blotting using LAPTM4B antibody (Left Panel) or GFP antibody (Right Panel). The black arrow designates the endogenous LAPTM4B, while the green arrow denotes the sfGFP-tagged LAPTM4B. **(D)**The endogenous labeled cell lines (L+C tag) and wild-type cell lines (WT) were transfected with siRNA against LAPTM4B or CD63, the protein levels were then determined by western blotting using CD63 antibody (Left Panel) or mCherry antibody (Right Panel). **(E)**The flow-process diagram of the drug screening experiments in the current study. Cells were seeded in 384-well plates using a robotic system and incubated with an oncology collection drug library comprising 527 compounds, including FDA-approved drugs and emerging investigational agents. The cells were then fixed and imaged using the PerkinElmer Opera Phenix high-content microscopy system. Automated analysis was employed to quantify the image signals, and to discover compounds targeting LAPTM4B without affecting CD63 levels. **(F)**The signal intensity of LAPTM4B-sfGFP and CD63-mCherry after drugs treatment were highlighted by dot plot. Blue dot indicates compounds that reduce LAPTM4B level, red dot indicates compounds that increase LAPTM4B level. Several PI3K inhibitors were highlighted. **(G)**The table lists top 5 compounds that decrease LAPTM4B levels, while not affects CD63 levels. **(H)**Cells were treated with the indicated compounds, LAPTM4B levels and CD63 levels were measured afterward by western blot. The black arrow indicates the CD63 band.

To this end, we generated a double knock-in reporter cell line expressing genomically GFP-tagged LAPTM4B and mCherry-tagged CD63 (**Fig. 7A**). LAPTM4B-GFP was used to assess drug-induced changes in expression and CD63-mCherry as a reference endosomal protein that should not be affected by the drug. Both proteins showed mainly similar endosomal/lysosomal localization (**Fig. 7B**), in agreement with previous studies 15,27, and CD63-mCherry was in addition partially expressed in the plasma membrane (**Fig. 7B**). The knock-in cell line was verified by the treatment of siRNAs targeting LAPTM4B or CD63 (**Fig. 7C-D**).

We next conducted a high-content drug screen incorporating automated cell seeding, compound administration, high-resolution imaging, and semi-automated image analysis (**Fig. 7E**) 38,39. The reporter cells were treated with a library of 527 FDA-approved and investigational drugs, and single-cell fluorescence intensity measurements were used to quantify protein expression (**Supplementary Table S4**). The screen revealed that drugs which selectively reduced LAPTM4B expression were mainly inhibitors of mTOR and PI3K,* e.g.* PTC-209, copanlisib, and gedatolisib (**Fig. 7F-G**). To validate these candidate drugs, we performed western blotting and confirmed that these compounds indeed reduce the protein level of LAPTM4B without affecting CD63 level (**Fig. 7H**).

### Suppressors of LAPTM4B expression enhance EGFR-TKI efficacy *in vitro* and *in vivo*

**Figure 8:**
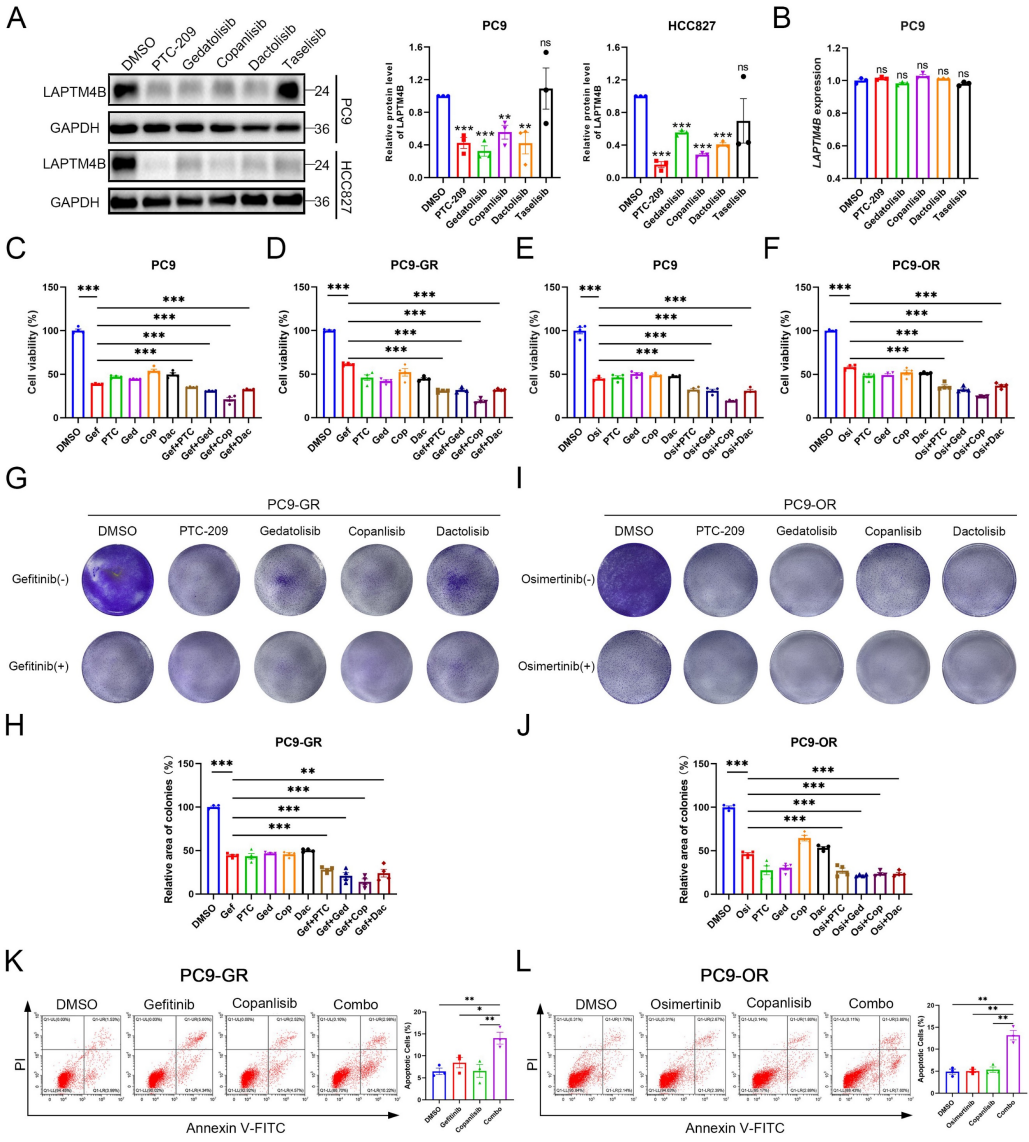
** Suppressors of LAPTM4B expression improve the treatment efficacy of EGFR-TKIs *in vitro.* (A)**PC9 and HCC827 cells were treated with suppressors of LAPTM4B expression, LAPTM4B levels were determined afterward by western blot. Quantification of at least three experiments. **(B)**PC9 cells were treated with suppressors of LAPTM4B expression, LAPTM4B transcript levels were measured by Q-PCR. **(C)**Cell viability in PC9 cells incubated with gefitinib, together with or without suppressors of LAPTM4B expression. Gef: Gefitinib; PTC: PTC-209; Ged: Gedatolisib; Cop: Copanlisib; Dac: Dactolisib. N=4. **(D)**Cell viability in PC9-GR cells incubated with gefitinib, together with or without suppressors of LAPTM4B expression. N=4. **(E)**Cell viability in PC9 cells incubated with osimertinib, together with or without suppressors of LAPTM4B expression. N=4. **(F)**Cell viability in PC9-OR cells incubated with osimertinib, together with or without suppressors of LAPTM4B expression. N=4. **(G)**In PC9-GR, colony formation experiments were employed to determine the cytotoxicity effect of LAPTM4B suppressors, with or without gefitinib. **(H)**Quantification of **(G)**. N=4. **(I)**In PC9-OR, colony formation experiments were employed to determine the cytotoxicity effect of LAPTM4B suppressors, with or without osimertinib. **(J)**Quantification of **(I)**. N=4. **(K)**PC9-GR cells were incubated with gefitinib, copanlisib, alone or in combination (Combo). Apoptotic cells (%) were then determined by flow cytometry. Left Panel: Representative FACS results; Right Panel: Statistical analysis of apoptotic cells percentage. * indicates p<0.05, ** indicates p<0.01. **(L)**PC9-OR cells were incubated with osimertinib, copanlisib, alone or combination. Apoptotic cells (%) were then determined by flow cytometry. Left Panel: Representative FACS results; Right Panel: Statistical analysis of apoptotic cells percentage. ** indicates p<0.01.

To explore the therapeutic potential of LAPTM4B-targeting compounds, we first evaluated candidate drugs in NSCLC cell lines harboring EGFR exon 19 deletions (PC9, HCC827). Treatment with PTC-209, copanlisib, gedatolisib, or dactolisib effectively reduced LAPTM4B protein levels (**Fig. 8A**), without significantly altering mRNA expression (**Fig. 8B**), suggesting these compounds mainly reduce LAPTM4B levels via regulating the protein stability. Subsequently, we determined the IC_50_ value of these compounds (**Supplementary Fig. S10A-D**), and assessed the effect of these suppressors in combination with EGFR-TKIs treatment in both wild-type (PC9) and resistant cell lines (PC9-GR and PC9-OR).

The combination of gefitinib with these compounds resulted in a significantly greater reduction in cell viability in PC9 cells compared to treatment with gefitinib alone (**Fig. 8C**), and the reduction was particularly pronounced in the resistant PC9-GR cells (**Fig. 8D**). Similarly, combination with osimertinib produced enhanced cytotoxicity in both parental and resistant PC9-OR cells (**Fig. 8E-F**). Accordingly, colony formation experiments further supported the synergistical effect of drug combination in term of inhibiting the growth of both PC9-GR cells (**Fig. 8G-H**) and PC9-OR cells (**Fig. 8I-J**). The similar results were observed in PC9 cells treated with gefitinib or osimertinib (**Supplementary Fig. S10E-H**).

Given its pronounced synergy, copanlisib was selected for further evaluation. Flow cytometry assays reported an enhanced cellular apoptosis rate upon the drug combination treatment of copanlisib with gefitinib (**Fig. 8K**), or with osimertinib (**Fig. 8L**), compared to single compound treatment. These findings were further supported by elevated levels of cleaved Caspase-3/7 in drug combination-treated PC9-GR and PC9-OR cells (**Supplementary Fig. S10I-J**).

Subsequently, we assessed the impact of copanlisib on the p-EGFR signaling pathway and found that copanlisib treatment attenuates p-EGFR signaling in PC9, PC9-GR, and PC9-OR cells, further supporting the crucial role of LAPTM4B in sustaining p-EGFR signaling (**Supplementary Fig. S10K**).

**Figure 9:**
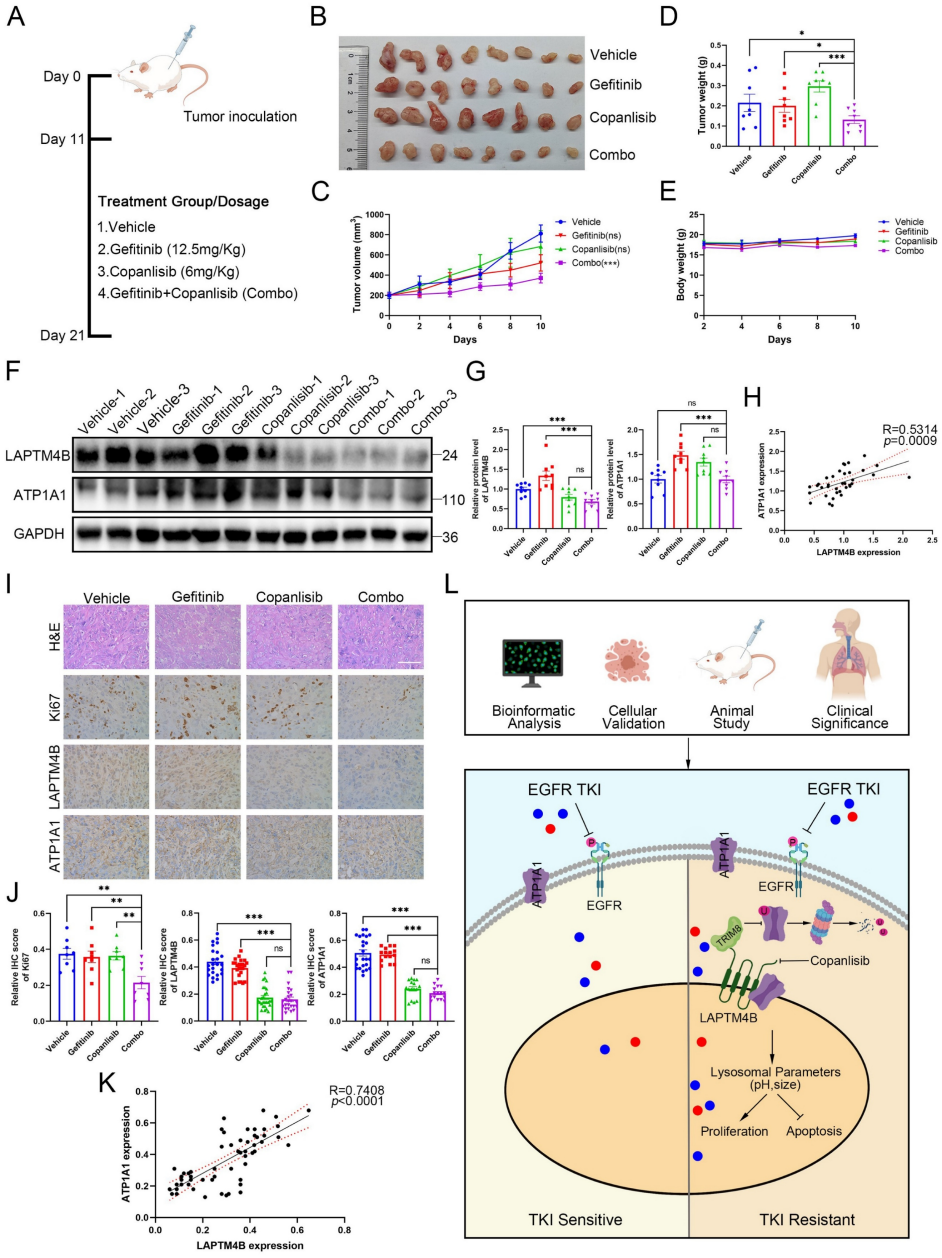
** Combined administration of copanlisib with gefitinib synergistically inhibits tumor growth in xenograft model bearing PC9-GR cells. (A)**Flowchart depicting the experimental design of the nude mice xenograft study. Female nude mice were injected with 1x10^7^ PC9-GR cells into the flanks. Once viable tumors had formed after 11 days, the mice were randomly divided into four subgroups and treated with gefitinib (12.5mg/Kg), copanlisib (6mg/Kg), gefitinib+copanlisib (Combo) or vesicle. The mice were sacrificed after 10 days of treatment. **(B)**Photographs of the tumors formed in the mice at the time of sacrifice. **(C)**Tumor volume in the mice with different treatments. Data presented as mean ± SEM. Tumor volume were calculated as: 1/2 Length*Width^2^. **(D)**Tumor weight (grams) in the mice with different treatments, top 8 tumor weight in each group were selected and analyzed. **(E)**Body weight (grams) of the mice with different treatments. **(F)**Western blotting analysis of LAPTM4B and ATP1A1 protein levels in mouse tumor tissue samples. **(G)**Quantification of Western blotting results from **(F)**. **(H)**Correlation between expression levels of LAPTM4B and ATP1A1 in mouse tumor samples measured by western blotting (R=0.5314, *p*=0.0009). (I)Immunohistochemistry (IHC) staining of Ki67, LAPTM4B, and ATP1A1 in mouse tumor tissue samples. Representative images from H&E staining and IHC staining. Scale bar: 100 µm. **(J)**Quantification of IHC results from **(I)**. ** indicates p<0.01, *** indicates p<0.001. **(K)**Correlation between expression levels of LAPTM4B and ATP1A1 in mouse tumor samples measured by immunohistochemistry (R=0.7408, *p*<0.0001). **(L)**Schematic diagram depicting the working model in the current study. LAPTM4B interacts with ATP1A1, stabilizes the protein by suppressing the TRIM8 mediated K63-ubiquitination and proteasomal degradation, further regulates lysosomal parameter and promotes the resistance to EGFR-TKIs. Suppressors of LAPTM4B expression synergistically enhance the inhibitory effect of EGFR-TKIs on NSCLC.

To further gain *in vivo* insight, we utilized copanlisib and gefitinib, alone or in combination, to assess the efficacy and tolerability of drug combination in the animal experiments. To this end, we subcutaneously implanted the drug-resistant cell line PC9-GR into 20 immunocompromised mice, and after 11 days when tumors became visually apparent, the mice were randomly divided into four groups and administered with vehicle, copanlisib (Intraperitoneally), gefitinib (Intragastrically), or the combination. The treatments continued for 10 days until the mice were subsequently euthanized (**Fig. 9A**). Notably, two mice succumbed during the treatment phase, and variations in tumor development were observed among the different experimental groups. We therefore selected the eight most substantial tumors for further analysis. Interestingly, the combination of drugs significantly reduced tumor growth compared to the vehicle and single agents (**Fig. 9B**). Consequently, the tumor volume and weight were less in the combination group than in the single drug or the vehicle group (**Fig. 9C-D**).

Despite the compounds used have been approved by the U.S. Food and Drug Administration (FDA), we still assessed the possible side effects of the drug combination. Mice in each treatment group did not experience any significant weight loss (**Fig. 9E**), and examination of major organs through pathological staining revealed no signs of toxicity (**Supplementary Fig. S11A**). These findings suggest that the drug treatments were well-tolerated with minimal side effects in our study.

Further examination of the tumor samples by western blotting revealed decreased LAPTM4B levels in mice tumor samples treated with “copanlisib” or “combination” (**Fig. 9F-G**). Interestingly, the expression of LAPTM4B positively correlated with the ATP1A1 levels in these tumor samples from nude mice (**Fig. 9H**). Moreover, these findings were supported by immunohistochemistry staining (**Fig. 9I-K**). Additionally, Ki67 staining in tumor tissues significantly reduced upon the drug combination treatment (**Fig. 9I-J**). Collectively, these data demonstrate that pharmacologic suppression of LAPTM4B enhances EGFR-TKI efficacy in NSCLC both *in vitro* and *in vivo*, with minimal toxicity. In summary, our study identifies LAPTM4B as a lysosomal regulator that promotes EGFR-TKI resistance in NSCLC. Mechanistically, LAPTM4B interacts with and recruits ATP1A1 to lysosome, stabilizes the protein via suppressing TRIM8-mediated K63-linked ubiquitination of ATP1A1. As a consequence, LAPTM4B promotes the lysosomal acidification and stimulates the activation of p-EGFR and downstream signaling pathway. Using an unbiased high-content drug screen, we identified several small molecules that selectively inhibit LAPTM4B, and demonstrated that combination treatment with copanlisib and EGFR-TKIs produces robust anti-tumor effects *in vitro* and *in vivo* with negligible side effects. These findings provide both mechanistic insight into EGFR-TKI resistance and a foundation for potential clinical translation (**Fig. 9L**).

## Discussion

Acquired resistance to EGFR-TKIs remains a major clinical challenge in the treatment of NSCLC, and extensive research has primarily focused on identifying secondary mutations within the EGFR gene as key drivers of resistance 50. Despite the development of next-generation EGFR-TKIs, resistance inevitably emerges, and the underlying mechanisms remain incompletely understood 50-53. In this study, we identify lysosomal dysregulation which specifically involves LAPTM4B and ATP1A1, as a previously unrecognized contributor to acquired resistance against both first- and third-generation EGFR-TKIs.

Lysosomes, traditionally regarded as degradative organelles 7, are now increasingly recognized as dynamic signaling hubs that regulate cellular metabolism, signal transduction, and therapy resistance in cancer 8. LAPTM4B has been implicated in tumor progression 11-17 and metabolic reprogramming 23-26 across multiple cancer types. In this study, we demonstrate that LAPTM4B is markedly upregulated in EGFR-mutant NSCLC, with further elevation observed in tumors from patients who have developed resistance to EGFR-TKI therapy. Functional analyses reveal that depletion of LAPTM4B sensitizes NSCLC cells to EGFR-TKIs and attenuates downstream phospho-EGFR signaling, highlighting its essential role in sustaining oncogenic signaling under targeted therapeutic pressure. These findings not only reinforce previous reports that LAPTM4B suppresses EGFR degradation and maintains downstream pathway activation 18,54, but also extend its relevance to the context of acquired resistance to EGFR-targeted therapies.

Mechanistically, we demonstrate that EGFR-TKIs induce LAPTM4B accumulation not via transcriptional upregulation, but through stabilization of the protein. This occurs through suppression of the E3 ubiquitin ligase WWP2, which we identify as a regulator of LAPTM4B turnover. This WWP2-LAPTM4B axis represents a feedback mechanism whereby EGFR-TKI treatment promotes LAPTM4B accumulation, thereby enabling adaptive resistance. While previous studies have linked LAPTM4B to resistance against chemotherapy 19,20, our data extend its role to targeted therapy and reveal a new post-translational layer of regulation in NSCLC.

Another interesting finding is the identification of lysosome-localized ATP1A1 as a functional partner of LAPTM4B in mediating EGFR-TKI resistance. Although ATP1A1 internalization and endocytosis have been reported previously 34-36, its lysosomal localization and functional contribution to lysosomal acidification in the context of drug resistance had not been described. Our data demonstrate that ATP1A1 cooperates with LAPTM4B to maintain lysosomal acidity, a critical determinant of EGFR-TKI efficacy. This introduces a novel mechanism in which lysosomal pH homeostasis—regulated by a LAPTM4B-ATP1A1 axis—drives resistance to EGFR-targeted therapies.

These findings expand the prevailing framework of EGFR-TKI resistance beyond the classical mutation-centric paradigm, introducing a lysosome-centered model of therapeutic adaptation. Rather than serving merely as a degradative endpoint, the lysosome emerges as a dynamic and actively regulated signaling hub that modulates drug response. While previous studies have predominantly linked lysosomal adaptation to chemotherapeutic drug sequestration or alterations in autophagic flux 8, we show here that lysosomal acidification per se is a critical determinant of EGFR-TKI efficacy. This process is tightly regulated by the cooperative action of LAPTM4B and ATP1A1. Our study highlights the lysosome as a dynamic and druggable hub in targeted therapy resistance.

Importantly, through unbiased high-content drug screening, we identified small-molecule inhibitors of LAPTM4B and demonstrated their synergistic effects with EGFR-TKIs in NSCLC models. These findings suggest that combinatorial targeting of LAPTM4B could enhance EGFR-TKI efficacy. Further preclinical studies using patient-derived xenograft (PDX) models and LAPTM4B knockout mice will be essential to evaluate the translational potential of this strategy. Moreover, structural studies of LAPTM4B-ATP1A1 interactions and the regulatory mechanisms governing lysosomal acidification may also reveal additional druggable targets and inform next-generation therapeutic designs.

In summary, our study establishes LAPTM4B as a pivotal effector of EGFR-TKI resistance via modulation of lysosomal acidification. Together with lysosomal ATP1A1, LAPTM4B forms a lysosomal regulatory axis that sustains oncogenic signaling under EGFR-TKI pressure. These findings not only illuminate a previously unrecognized mechanism of resistance but also suggest new strategies for overcoming therapeutic failure through lysosome-targeted interventions.

## Supplementary Material

Supplementary methods, figures and tables 1-2.

Supplementary table 3.

Supplementary table 4.

## Abbreviations

NSCLC: Non-small cell lung cancer
LAPTM4B: Lysosome Associated Protein Transmembrane 4B
EGFR: Epidermal Growth Factor Receptor
TKIs: Tyrosine Kinase Inhibitors
KO: knockout
