# Supplementary material for: LAPTM4B Confers Resistance to EGFR-TKIs by Suppressing the Proteasomal Degradation of ATP1A1 in Non-small Cell Lung Cancer
Source: Int J Biol Sci. 2026 Jan 1;22(1):1–24. doi: 10.7150/ijbs.115365 (PMC12681703; doi:10.7150/ijbs.115365)
Supplement: Supplementary file 1 — Supplementary methods, figures and tables 1-2. [file ijbsv22p0001s1.pdf]

Supplementary Figure

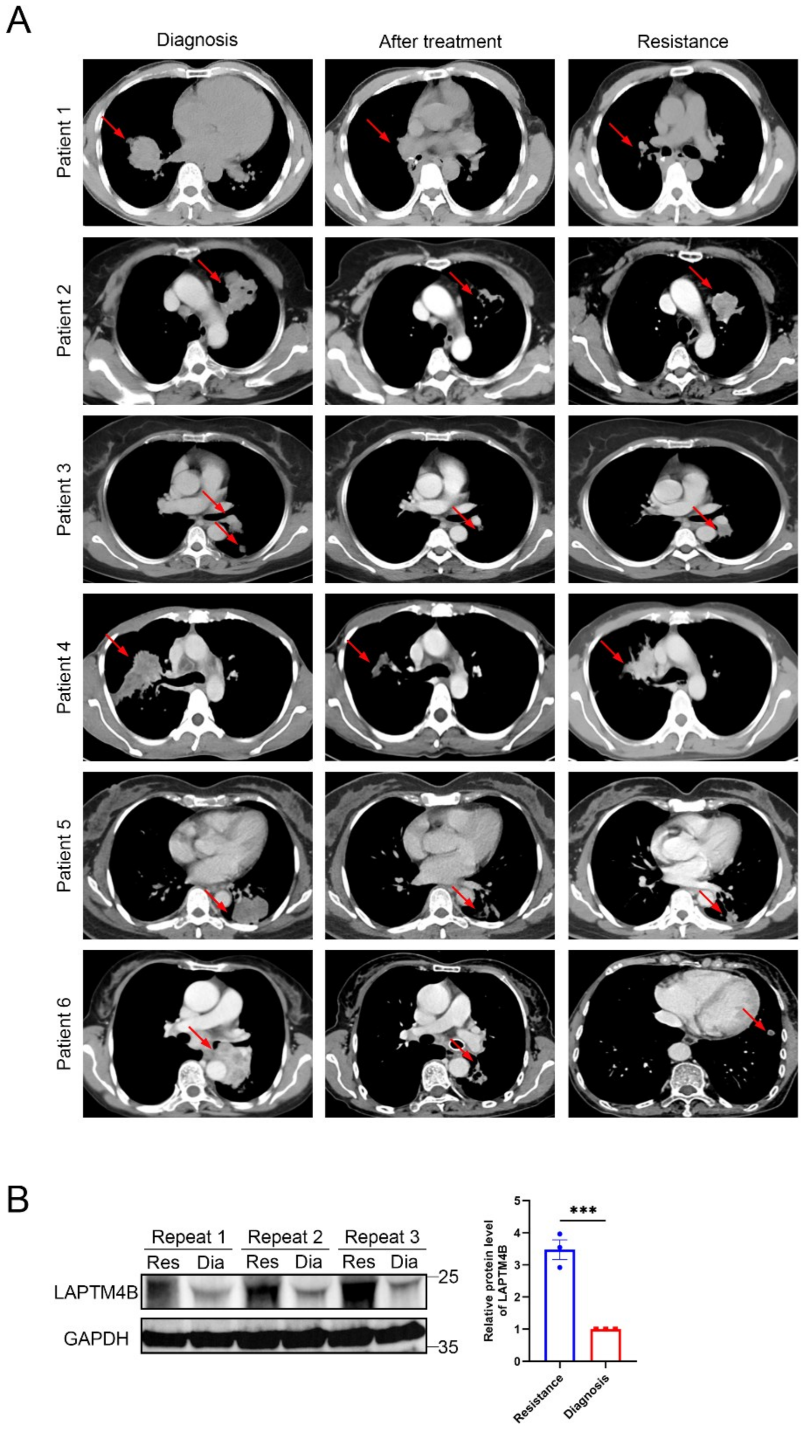

Supplementary Figure S1: LAPTM4B is upregulated in EGFR-TKI resistant NSCLC patients

(A) Computed tomography (CT) images of lung tumors from EGFR-mutant

NSCLC patients at diagnosis, following initial EGFR-TKI treatment, and after the development of clinical resistance. Red arrows indicate the tumor localization.

(B) Western blot analysis comparing LAPTM4B protein levels in resistant (Res) versus diagnostic (Dia) tumor tissues. Quantification of (H). N=3 technical repeats.

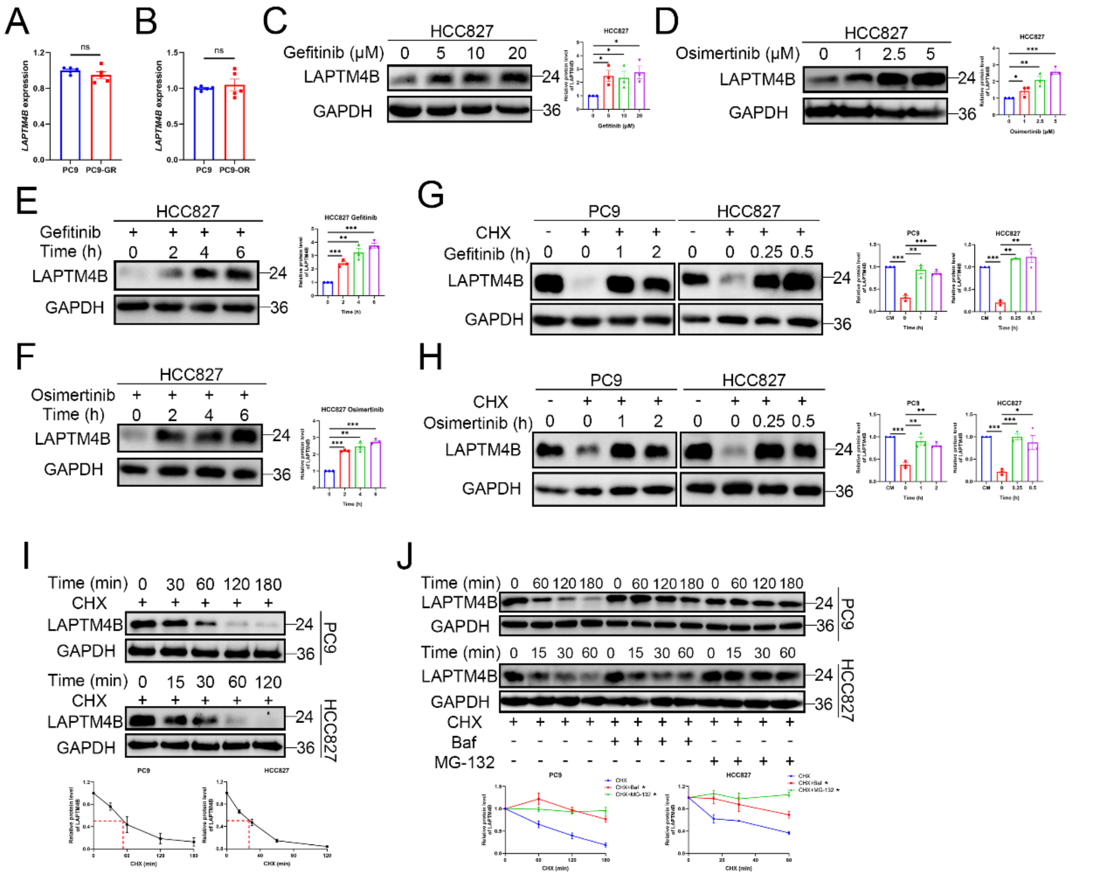

Supplementary Figure S2: EGFR-TKIs upregulate LAPTM4B by enhancing protein stability

(A) Quantitative PCR analysis of LAPTM4B mRNA levels in PC9 and PC9-GR cells. Quantification of n=5 experiments, presented as mean ± SEM, data normalized to "PC9".

(B) Quantitative PCR analysis of LAPTM4B mRNA levels in PC9 and PC9-OR cells. Quantification of n=5 experiments, presented as mean ± SEM, data normalized to "PC9".

(C) HCC827 cells were treated with gefitinib for 6 h at the indicated concentration, and the expression of LAPTM4B was measured by western blotting. Quantification of n=3 experiments.

(D) HCC827 cells were treated with osimertinib for 6 h at the indicated

concentration, and the expression of LAPTM4B was measured by western blotting. Quantification of n=3 experiments.

(E) HCC827 cells were treated with 10 μM gefitinib for the indicated time, and the expression of LAPTM4B was measured by western blotting. Quantification of n=3 experiments.

(F) HCC827 cells were treated with 2.5 μM osimertinib for the indicated time, and the expression of LAPTM4B was measured by western blotting. Quantification of n=3 experiments.

(G) PC9 and HCC827 cells were treated with 50 μg/mL cycloheximide (CHX) together with 10 μM gefitinib for the indicated time, and the expression of LAPTM4B was measured by western blotting. Quantification of n=3 experiments. CM: Complete Medium.

(H) PC9 and HCC827 cells were treated with CHX together with 2.5 μM Osimertinib for the indicated time, and the expression of LAPTM4B was measured by western blotting. Quantification of n=3 experiments. CM: Complete Medium.

(I) PC9 and HCC827 cells were treated with CHX for the indicated time, and the expression of LAPTM4B was measured by western blotting.

(J) PC9 and HCC827 cells were treated with 1 μmol/L bafilomycin-A1 (BafA1) or 20 μmol/L MG-132, together with 50 μg/mL CHX, for the indicated time, and the expression of LAPTM4B was measured by western blotting.

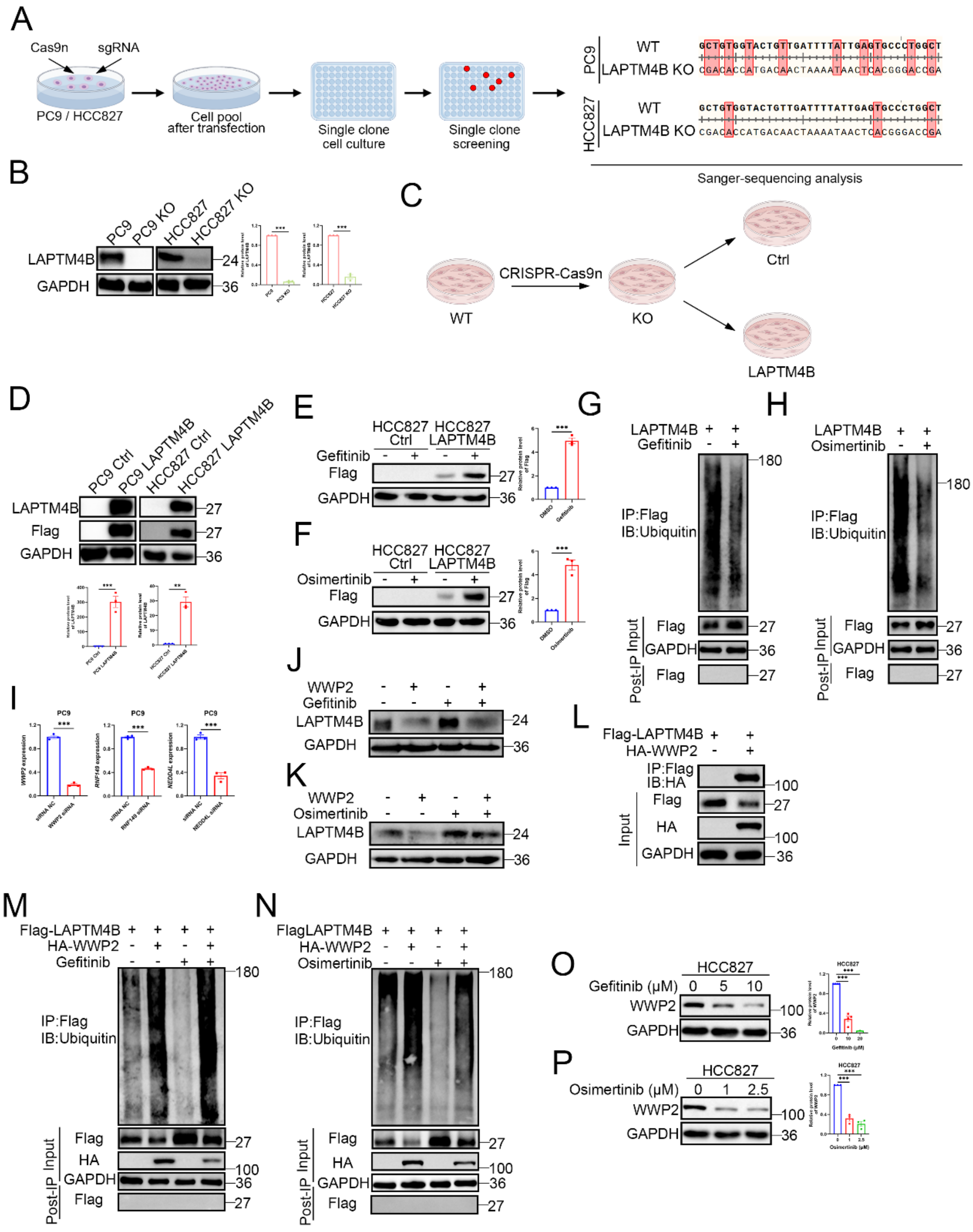

Supplementary Figure S3. Gefitinib and osimertinib upregulate LAPTM4B by suppressing WWP2-mediated proteasomal degradation.

(A) Generation of LAPTM4B knockout (KO) cell lines by CRISPR/Cas9n. Cells were transfected with Cas9n and sgRNAs targeting LAPTM4B, followed by puromycin selection and single-cell cloning. Representative Sanger

sequencing results show the mutations in LAPTM4B-depleted cells, particularly evident in PC9 KO clones.

(B) Western blot analysis comparing LAPTM4B protein levels in wild-type (WT) versus LAPTM4B knockout (KO) PC9 and HCC827 cells. Quantification of n=3 experiments, mean ± SEM.

(C) Schematic workflow for generating LAPTM4B stably expressing cells. LAPTM4B KO lines were derived from WT cells, and LAPTM4B was subsequently re-expressed in the KO background to establish stable rescue lines. Empty vector–transfected cells served as controls (Ctrl).

(D) Western blot analysis of LAPTM4B expression in LAPTM4B stably expressing PC9 or HCC827 cells, compared to control cells, using anti-LAPTM4B and anti-Flag antibodies.

(E) HCC827 Ctrl and HCC827 LAPTM4B cells were treated with 10 μM gefitinib for 6 h, and the expression of Flag-LAPTM4B was measured by western blotting.

(F) HCC827 Ctrl and HCC827 LAPTM4B cells were treated with 2.5 μM osimertinib for 6 h, and the expression of Flag-LAPTM4B was measured by western blotting.

(G) Flag-tagged LAPTM4B stably expressing HCC827 cells were treated with 10 μM gefitinib or DMSO for 6 h. Immunoprecipitation of the cell lysate using Flag antibody, followed by immunoblotting with Ubiquitin antibody.

(H) Flag-tagged LAPTM4B stably expressing HCC827 cells were treated with 2.5 μM osimertinib or DMSO for 6 h. Immunoprecipitation of the cell lysate using Flag antibody, followed by immunoblotting with Ubiquitin antibody.

(I) PC9 cells were transfected with indicated siRNA, the mRNA expression of WWP2, RNF149, and NEDD4L were measured by Q-PCR.

(J) HCC827 cells were overexpressed with WWP2, treated with 10 μM gefitinib or DMSO for 6 h. The expression of LAPTM4B was measured by western blotting.

(K) HCC827 cells were overexpressed with WWP2, treated with 2.5 μM osimertinib or DMSO for 6 h. The expression of LAPTM4B was measured by western blotting.

(L) Flag-tagged LAPTM4B stably expressing HCC827 cells were transfected with HA-tagged WWP2. Cell lysates were immunoprecipitated with anti-Flag beads and analyzed by immunoblotting with an anti-HA antibody.

(M) Flag-tagged LAPTM4B stably expressing HCC827 cells were transfected with or without HA-tagged WWP2, and afterwards treated with 10 μM gefitinib or DMSO for 6 h. Immunoprecipitation of the cell lysate using Flag antibody, followed by immunoblotting with Ubiquitin antibody.

(N) Flag-tagged LAPTM4B stably expressing HCC827 cells were transfected with or without HA-tagged WWP2, and afterwards treated with 2.5 μM osimertinib or DMSO for 6 h. Immunoprecipitation of the cell lysate using Flag antibody, followed by immunoblotting with Ubiquitin antibody.

(O) HCC827 cells were treated with gefitinib for 6 h at the indicated concentration, and the expression of WWP2 was measured by western blotting. Quantification of n=4 experiments.

(P) HCC827 cells were treated with osimertinib for 6 h at the indicated concentration, and the expression of WWP2 was measured by western blotting. Quantification of n=3 experiments.

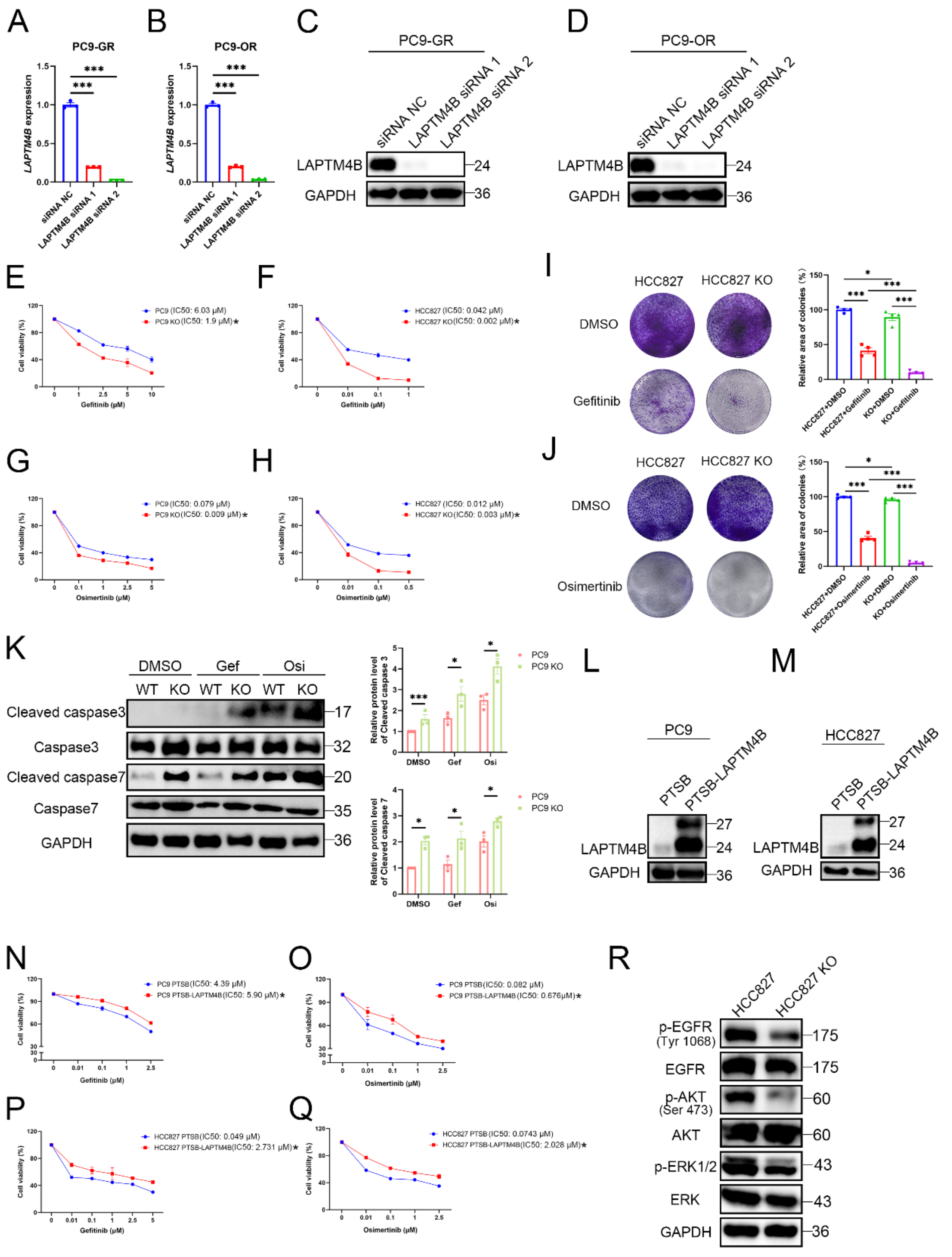

Supplementary Figure S4: LAPTM4B is critical for EGFR-TKIs resistance and maintenance of p-EGFR signaling

(A) PC9-GR cells were transfected with siRNAs against LAPTM4B, cells were harvested and followed by Q-PCR measuring LAPTM4B mRNA expression.

(B) PC9-OR cells were transfected with siRNAs against LAPTM4B, cells were

harvested and followed by Q-PCR measuring LAPTM4B mRNA expression.

(C) PC9-GR cells were transfected with siRNAs against LAPTM4B, cells were harvested 72 h afterwards and followed by western blotting measuring LAPTM4B levels.

(D) PC9-OR cells were transfected with siRNAs against LAPTM4B, cells were harvested 72 h afterwards and followed by western blotting measuring LAPTM4B levels.

(E) Cell viability in WT and LAPTM4B KO PC9 cells incubated with gefitinib. Quantification of n=3 experiments.

(F) Cell viability in WT and LAPTM4B KO HCC827 cells incubated with gefitinib. Quantification of n=3 experiments.

(G) Cell viability in WT and LAPTM4B KO PC9 cells incubated with osimertinib. N=3.

(H) Cell viability in WT and LAPTM4B KO HCC827 cells incubated with osimertinib. Quantification of n=3 experiments.

(I) 3×10^4 WT and LAPTM4B KO HCC827 cells were seeded into a 12-well plate, treated with gefitinib or DMSO, and cultured at 37°C for 8 days and change the medium every three days. Afterwards, the cells were fixed with 4% polyformaldehyde, stained with crystal violet, and subsequently imaged and quantified. Left panel: representative experiment. Right panel: quantification of n=4 experiments, mean ± SEM.

(J) 3×10^4 WT and LAPTM4B KO HCC827 cells were seeded into a 12-well plate, treated with osimertinib or DMSO, and cultured at 37°C for 8 days and change the medium every three days. Afterwards, the cells were fixed with 4% polyformaldehyde, stained with crystal violet, and subsequently imaged and quantified. Left panel: representative experiment. Right panel: quantification of n=4 experiments, mean ± SEM.

(K) Immunoblot analysis of cleaved and total caspase-3/7 in WT and LAPTM4B KO PC9 cells treated with 10 μM gefitinib or 1 μM osimertinib for 24 h. Left panel: representative results. Right panel: Quantification of n=3 experiments, mean ± SEM.

(L) PC9 cells were transiently transfected with PTSB-LAPTM4B or the empty vector (PTSB), and LAPTM4B protein levels were assessed by western blotting.

(M) HCC827 cells were transiently transfected with PTSB-LAPTM4B or the empty vector (PTSB), and LAPTM4B protein levels were assessed by western blotting.

(N) Cell viability in PC9 PTSB and PC9 PTSB-LAPTM4B cells incubated with gefitinib for 48h. Quantification of n=3 experiments.

(O) Cell viability in PC9 PTSB and PC9 PTSB-LAPTM4B cells incubated with osimertinib for 48h. Quantification of n=3 experiments.

(P) Cell viability in HCC827 PTSB and HCC827 PTSB-LAPTM4B cells incubated with gefitinib for 48h. Quantification of n=3 experiments.

(Q) Cell viability in HCC827 PTSB and HCC827 PTSB-LAPTM4B cells incubated with osimertinib for 48h. Quantification of n=3 experiments.

(R) Western blot analysis of p-EGFR, p-AKT, p-ERK expression levels in HCC827 and HCC827 KO cells.

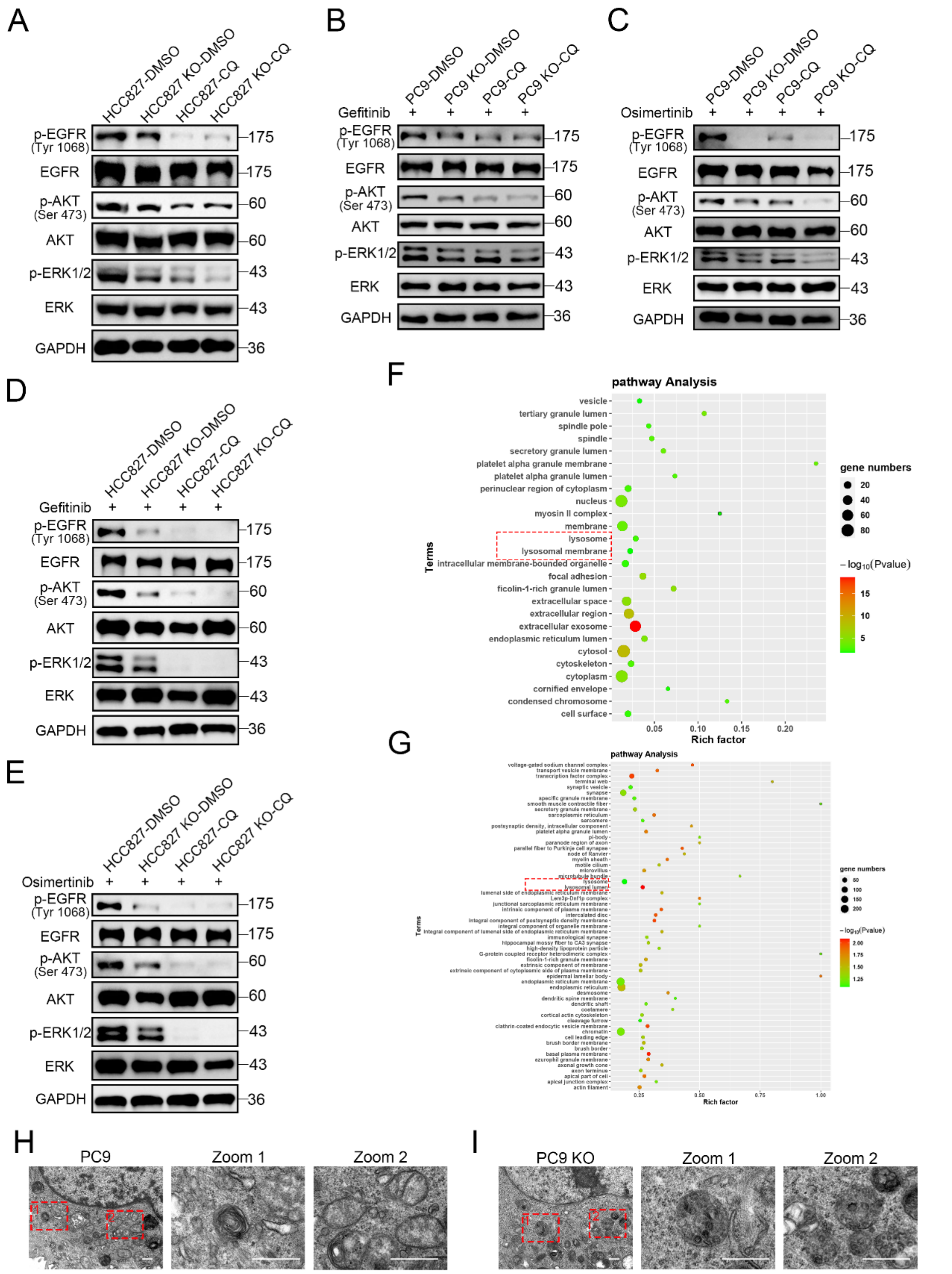

Supplementary Figure S5: LAPTM4B-mediated activation of p-EGFR signaling depends on lysosomal function

(A) Immunoblotting of p-EGFR, p-AKT, p-ERK in HCC827 and HCC827 KO cells, treated with chloroquine (CQ) or DMSO for 48h.

(B) Immunoblotting of p-EGFR, p-AKT, p-ERK in PC9 and PC9 KO cells, treated with chloroquine (CQ) or DMSO for 48h. All cells were treated with 2.5 μM gefitinib for 48 h.

(C) Immunoblotting of p-EGFR, p-AKT, p-ERK in PC9 and PC9 KO cells, treated with chloroquine (CQ) or DMSO for 48h. All cells were treated with 1 μM osimertinib for 48 h.

(D) Immunoblotting of p-EGFR, p-AKT, p-ERK in HCC827 and HCC827 KO cells, treated with chloroquine (CQ) or DMSO for 48h. All cells were treated with 0.1 μM gefitinib for 48 h.

(E) Immunoblotting of p-EGFR, p-AKT, p-ERK in HCC827 and HCC827 KO cells, treated with chloroquine (CQ) or DMSO for 48h. All cells were treated with 0.1 μM osimertinib for 48 h.

(F) Analysis the single-cell RNA sequencing data revealed the enrichment of "Lysosome" and "Lysosome membrane" in the cell population with TKIs resistance.

(G) Analysis the single-cell RNA sequencing data from GSE database (GSE#121634), the plot showed the enrichment of "Lysosome" and "Lysosome lumen" in the cell population with TKIs resistance.

(H) Representative image of the lysosome ultrastructure in PC9 cells. Scale bar: 0.5 μm. The regions in the dashed red box are amplified in the right panel.

(I) Representative image of the lysosome ultrastructure in LAPTM4B KO PC9 cells. Scale bar: 0.5 μm. The regions in the dashed red box are amplified in the right panel.

A

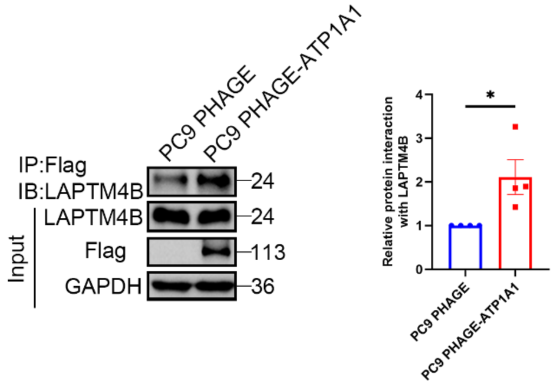

Supplementary Figure S6: LAPTM4B interacts with ATP1A1

(A) PC9 cells were transiently transfected with Flag-tagged ATP1A1 or empty vector (PHAGE). Cell lysates were subjected to immunoprecipitation with anti-Flag magnetic beads, followed by immunoblotting with anti-LAPTM4B antibody. Left panel: representative results. Right panel: Quantification of n=4 experiments， mean ± SEM.

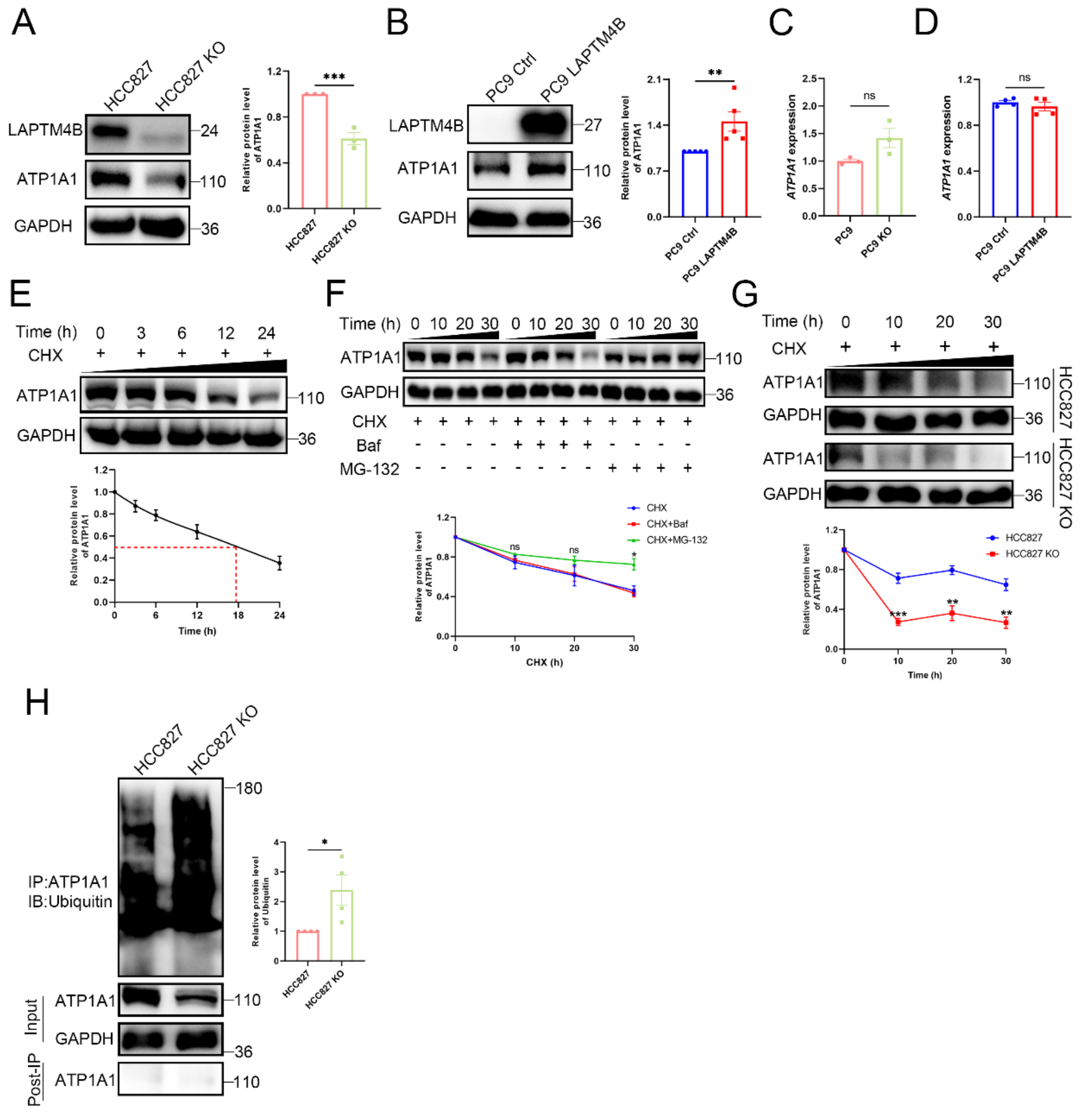

Supplementary Figure S7: LAPTM4B stabilized ATP1A1 via suppressing the ubiquitination-proteasomal degradation

(A) Western blotting analysis of ATP1A1 and LAPTM4B protein levels in LAPTM4B-depleted HCC827 cells. Left panel: Representative experiment. Right panel: Quantification of n=3 experiments, presented as mean ± SEM, *p*(HCC827, HCC827 KO)=0.0009.

(B) Western blotting analysis of ATP1A1 and LAPTM4B protein levels in LAPTM4B stably expressing PC9 and the control cells. Left panel:

Representative experiment. Right panel: Quantification of n=3 experiments, presented as mean ± SEM, *p*(Ctrl, LAPTM4B)=0.0009.

(C) Quantification of ATP1A1 mRNA expression in WT and LAPTM4B KO PC9 cells measured by Q-PCR. Quantification of n=3 experiments, presented as mean ± SEM, data normalized to "PC9".

(D) Quantification of ATP1A1 mRNA expression in LAPTM4B stably expressing PC9 and the control cells measured by Q-PCR. Quantification of n=4 experiments, presented as mean ± SEM, data normalized to "PC9 Ctrl".

(E) Western blot analysis of ATP1A1 protein levels in PC9 cells treated with 50 μg/mL cycloheximide (CHX) for the indicated times. Upper panel: Representative experiment. Lower panel: Quantification of n=3 experiments, presented as mean ± SEM. The red dashed line indicates the time point when half of the endogenous ATP1A1 has been degraded.

(F) Western blot analysis of ATP1A1 protein levels in PC9 cells treated with 1 μmol/L bafilomycin-A1 (BafA1) or 20 μmol/L MG-132, together with 50 μg/mL CHX for the indicated times. Upper panel: Representative experiment. Lower panel: Quantification of n=3 experiments, presented as mean ± SEM. *p*(CHX, CHX+MG-132_30 h)=0.0117.

(G) Western blot analysis of ATP1A1 protein levels in WT and LAPTM4B KO HCC827 cells treated with 50 μg/mL CHX for the indicated times. Upper panel: Representative experiment. Lower panel: Quantification of n=3 experiments, presented as mean ± SEM. *p*(HCC827, HCC827 KO_10 h)=0.0002. *p*(HCC827, HCC827 KO_20 h)=0.0012. *p*(HCC827, HCC827 KO_30 h)=0.0018.

(H) Immunoprecipitation of WT and LAPTM4B KO HCC827 cells using anti-ATP1A1 antibody, followed by immunoblotting with Ubiquitin antibody. Left panel: Representative experiment. Right panel: Quantification of n=4

experiments, mean ± SEM, data normalized to " HCC287". *p*(HCC287, HCC287 KO)=0.0177.

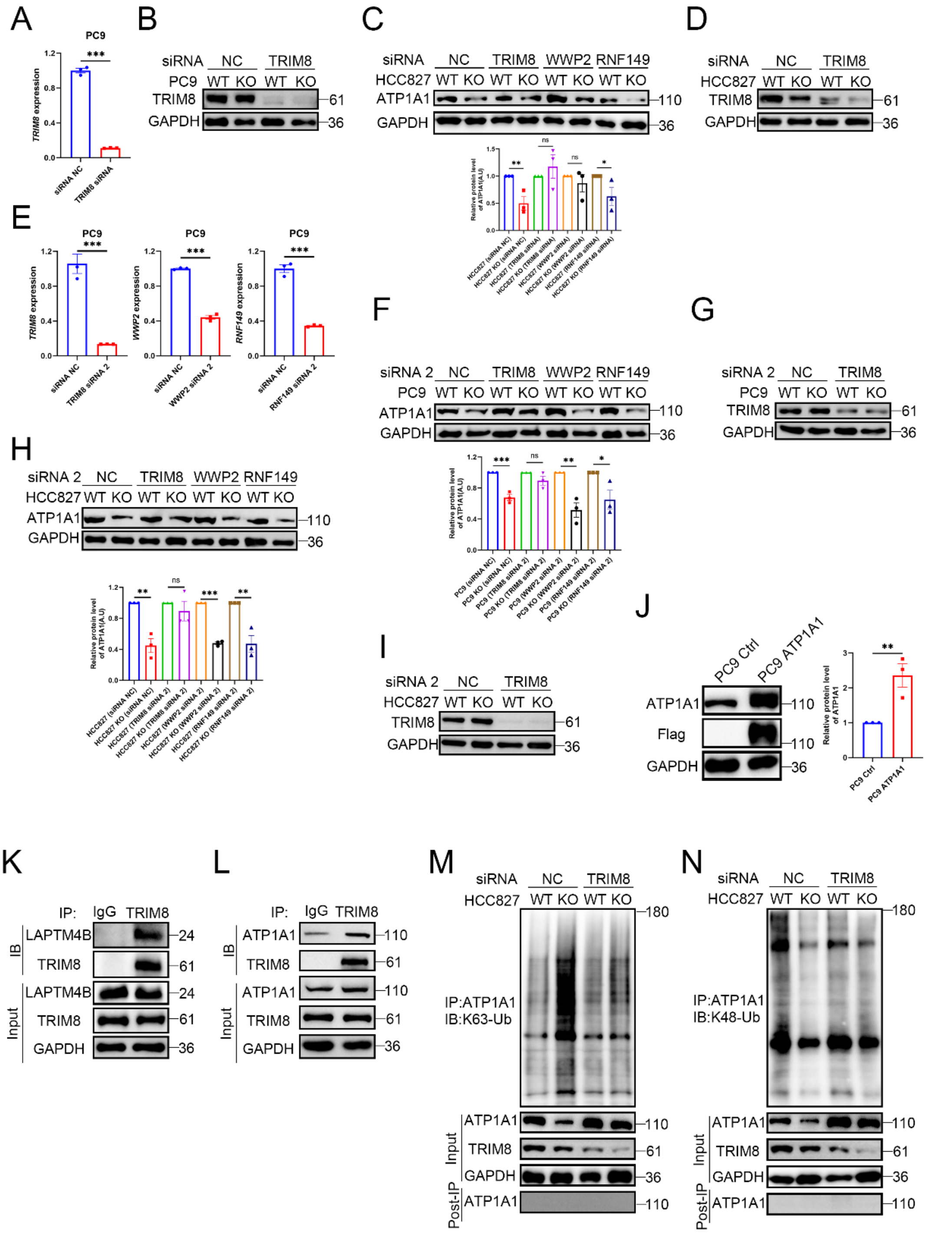

Supplementary Figure S8: TRIM8 mediated K63-linked ubiquitination is the critical mechanism of LAPTM4B regulating ATP1A1 protein stability

(A) Q-PCR analysis of TRIM8 mRNA levels following siRNA transfection in PC9 cells.

(B) Immunoblot analysis of TRIM8 protein levels in PC9 cells and PC9 KO cells transfected with TRIM8 siRNA.

(C) Immunoblot analysis of ATP1A1 protein levels in WT and LAPTM4B KO HCC827 cells transfected with the indicated siRNA. Quantification of n=3 experiments, presented as mean ± SEM. * $p<0.05$, ** $p<0.01$.

(D) Immunoblot analysis of TRIM8 protein levels in WT and LAPTM4B KO HCC827 cells transfected with TRIM8 siRNA.

(E) Q-PCR validation of a second independent siRNA targeting TRIM8, WWP2, or RNF149.

(F) Immunoblot analysis of ATP1A1 protein levels in WT and LAPTM4B KO PC9 cells transfected with the second independent siRNAs as indicated. Quantification of n=3 experiments, presented as mean ± SEM. * $p<0.05$, ** $p<0.01$, *** $p<0.001$

(G) Immunoblot analysis of TRIM8 protein levels in WT and LAPTM4B KO PC9 cells transfected with the second TRIM8 siRNA.

(H) Immunoblot analysis of ATP1A1 protein levels in WT and LAPTM4B KO HCC827 cells transfected with the second independent siRNAs. Quantification of n=3 experiments, presented as mean ± SEM. * $p<0.05$, ** $p<0.01$.

(I) Immunoblot analysis of TRIM8 protein levels in WT and LAPTM4B KO HCC827 cells transfected with the second TRIM8 siRNA.

(J) Flag tagged ATP1A1 stably expressing cells were generated from LAPTM4B KO background. Western blotting analysis were used to measure protein levels of ATP1A1 and Flag. Left panel: Representative experiment. Right panel: Quantification of n=3 experiments, presented as mean ± SEM, p(Ctrl, ATP1A1)=0.0076.

(K) PC9 cells were immunoprecipitated with TRIM8 antibody or IgG antibody, followed by immunoblotting with LAPTM4B antibody.

(L) PC9 cells were immunoprecipitated with TRIM8 antibody or IgG antibody, followed by immunoblotting with ATP1A1 antibody.

(M) WT and LAPTM4B KO HCC827 cells were transfected with indicated siRNA. Immunoprecipitation of the cell lysate using ATP1A1 antibody, followed by immunoblotting with K63-linkage specific polyubiquitin antibody.

(N) WT and LAPTM4B KO HCC827 cells were transfected with indicated siRNA. Immunoprecipitation of the cell lysate using ATP1A1 antibody, followed by immunoblotting with K48-linkage specific polyubiquitin antibody.

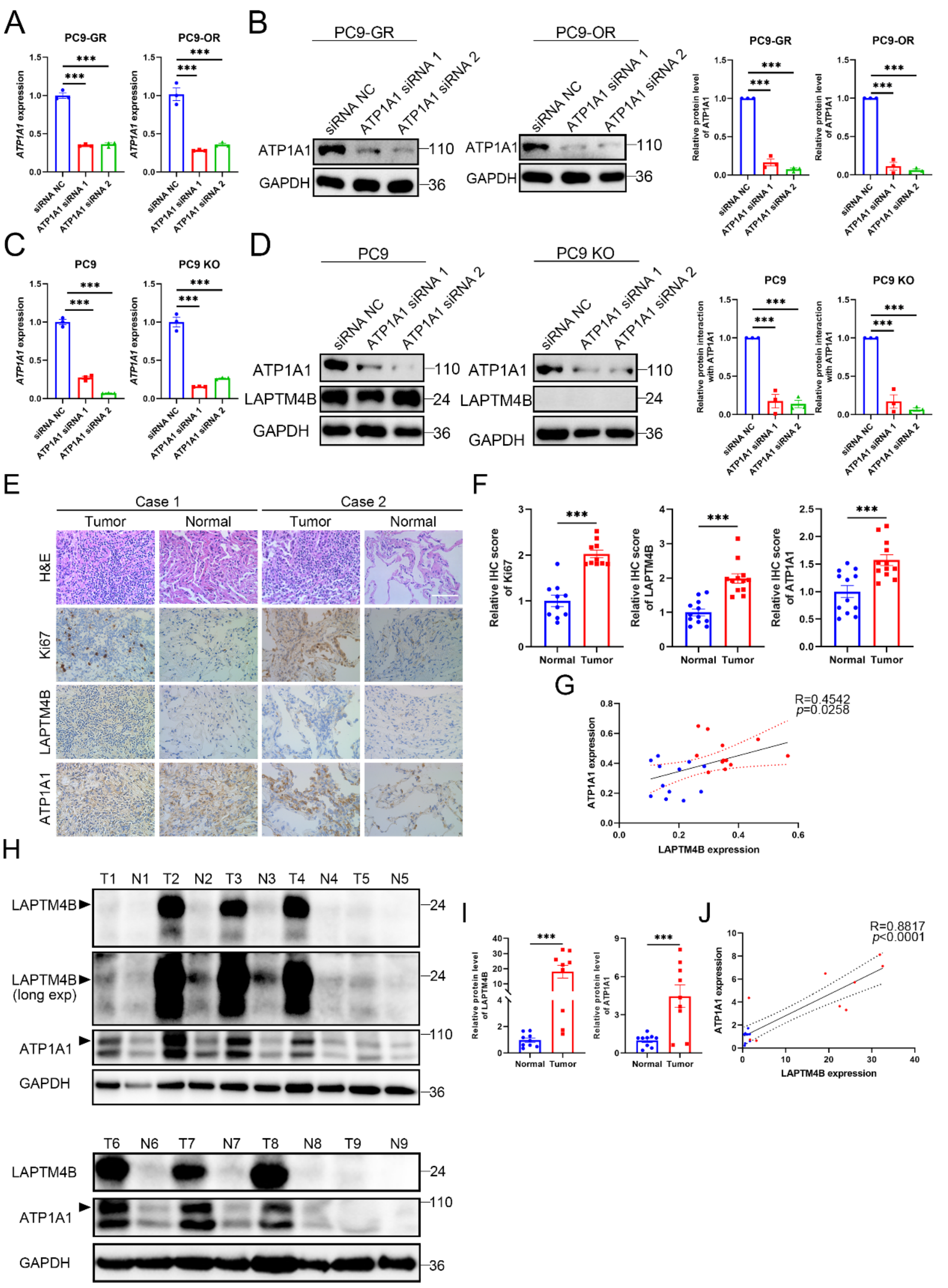

Supplementary Figure S9: ATP1A1 is crucial for LAPTM4B-mediated EGFR-TKIs resistance

(A) Q-PCR analysis of ATP1A1 mRNA levels in PC9-GR and PC9-OR cells

transfected with control siRNA or two independent siRNAs targeting ATP1A1. Quantification of n=3 experiments, presented as mean ± SEM.

(B) Immunoblot analysis of ATP1A1 protein levels in PC9-GR and PC9-OR cells transfected with ATP1A1 siRNAs. Left panel: Representative experiment. Right panel: Quantification of n=3 experiments, presented as mean ± SEM.

(C) Q-PCR analysis of ATP1A1 expression in PC9 cells and LAPTM4B KO PC9 cells following siRNA-mediated ATP1A1 knockdown. Quantification of n=3 experiments, presented as mean ± SEM.

(D) Immunoblot analysis of ATP1A1 protein levels in PC9 cells and LAPTM4B KO PC9 following ATP1A1 siRNA transfection. Left panel: Representative experiment. Right panel: Quantification of n=3 experiments, presented as mean ± SEM.

(E) Immunohistochemistry staining of Ki67, LAPTM4B and ATP1A1 in NSCLC tumor tissue and adjacent normal tissues. Scale bar: 100 μm.

(F) Quantification of IHC results from 12 patients. For each staining, at least five images per sample were evaluated and quantified.

(G) Correlation between expression levels of LAPTM4B and ATP1A1 in patients' tissue samples (R=0.4542, p=0.0258), based on the results from IHC staining. The red color represents "tumor tissue", blue color represents "adjacent normal tissue".

(H) Western blotting analysis of LAPTM4B and ATP1A1 levels in tumor tissue and adjacent normal tissues from collected NSCLC surgery samples. T: Tumor tissue; N: Adjacent normal tissue. The black arrow indicates the band of LAPTM4B or ATP1A1.

(I) Quantification of the data were from 9 pairs of NSCLC tissues.

(J) Correlation between expression levels of LAPTM4B and ATP1A1 in patients' tissue samples (R=0.8817, p<0.0001), based on the results from western

blotting. The red color represents “NSCLC tumor tissue”, blue color represents “adjacent normal tissue”.

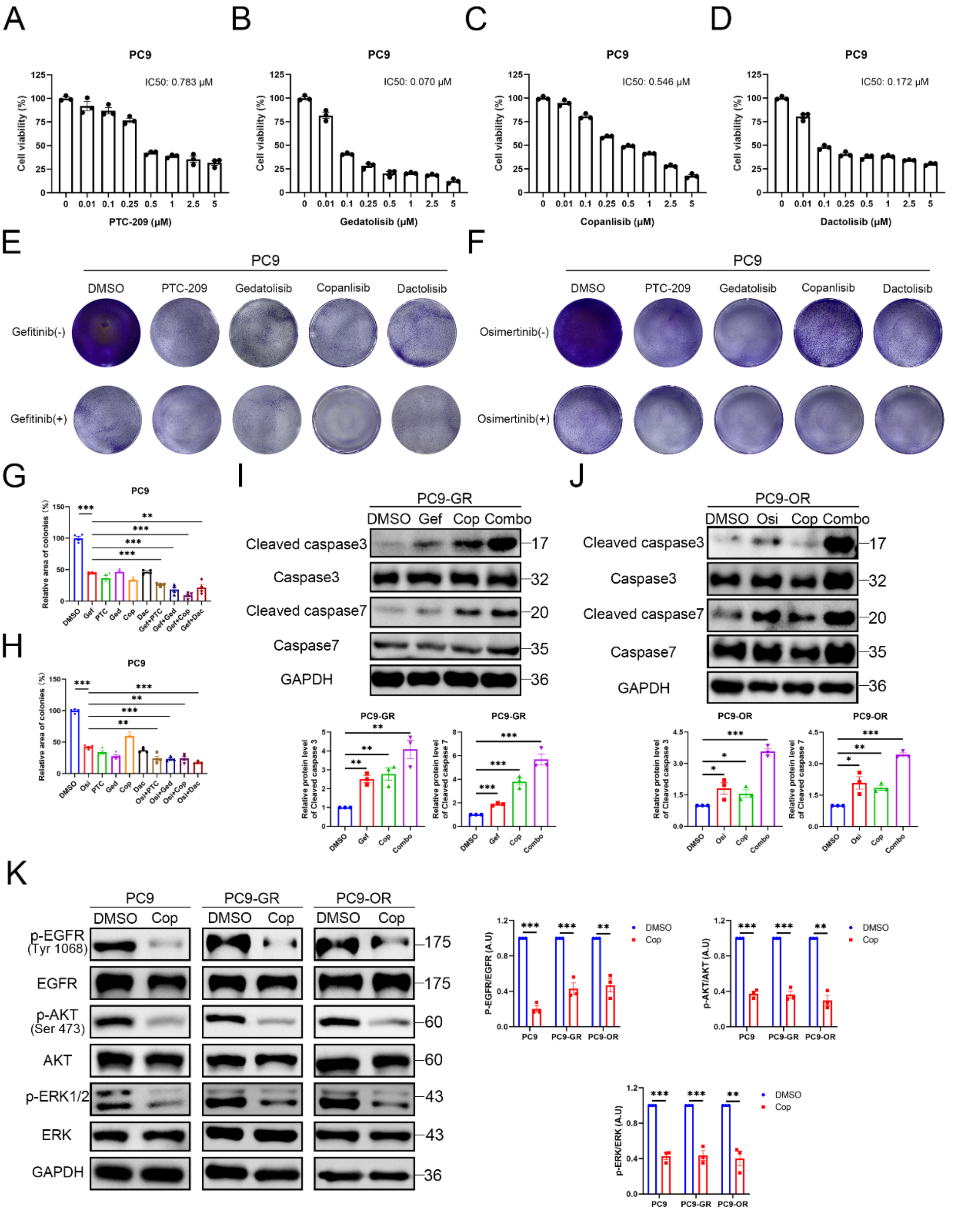

Supplementary Figure S10: Suppressors of LAPTM4B expression improve the treatment efficacy of EGFR-TKIs

(A) Cell viability in PC9 cells incubated with PTC-209 at the indicated concentrations for 48h. Quantification of at least three experiments, presented as mean ± SEM.

(B) Cell viability in PC9 cells incubated with gedatolisib at the indicated concentrations for 48h. Quantification of at least three experiments, presented as mean ± SEM.

(C) Cell viability in PC9 cells incubated with copanlisib at the indicated concentrations for 48h. Quantification of at least three experiments, presented as mean ± SEM.

(D) Cell viability in PC9 cells incubated with dactolisib at the indicated concentrations for 48h. Quantification of at least three experiments, presented as mean ± SEM.

(E) In PC9 cells, colony formation experiments were employed to determine the cytotoxicity effect of LAPTM4B suppressors, with or without gefitinib.

(F) In PC9 cells, colony formation experiments were employed to determine the cytotoxicity effect of LAPTM4B suppressors, with or without osimertinib. N=4.

(G) Quantification of colony formation experiments in (E). N=4.

(H) Quantification of colony formation experiments in (F). N=4.

(I) Western blot analysis of cleaved and total caspase-3/7 in PC9-GR cells treated with 10 μM gefitinib, with or without 0.5 μM copanlisib, for 24 h. Quantification of at least three experiments.

(J) Western blot analysis of cleaved and total caspase-3/7 in PC9-OR cells treated with 1 μM osimertinib, with or without 0.5 μM copanlisib, for 24 h. Quantification of at least three experiments.

(K) Immunoblot analysis of p-EGFR, p-AKT, and p-ERK in PC9, PC9-GR, and PC9-OR cells treated with 0.5 μM copanlisib for 24 h. Left panel: representative results. Right panel, Quantification of at least three experiments.

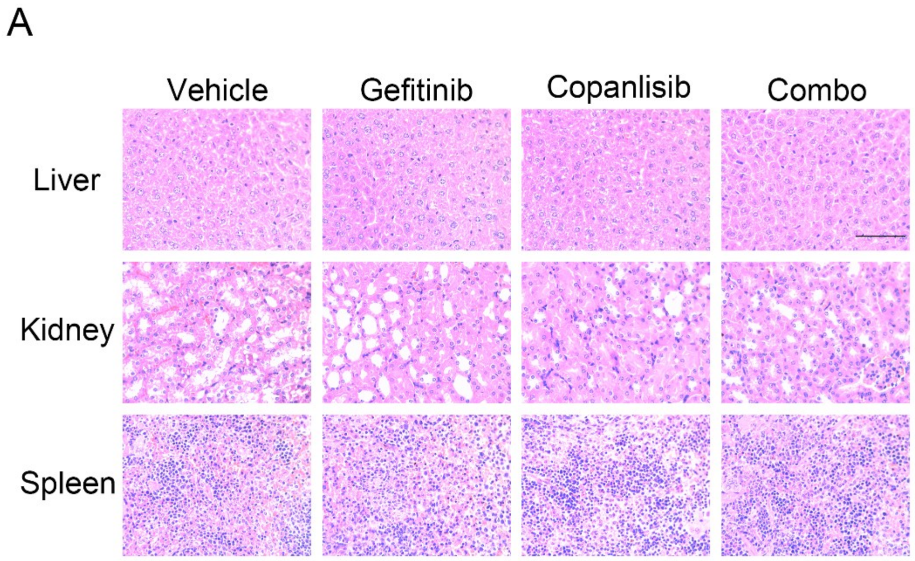

Supplementary Figure S11: No potential damage on major mice organs after LAPTM4B inhibitors treatment

(A) Female nude mice were injected with $1x10^7$ PC9-GR cells into the flanks. Once viable tumors, the mice were randomly divided into four subgroups and treated with Gefitinib, Copanlisib, Gefitinib+Copanlisib (Combo) or vesicle. The mice were sacrificed after 10 days of treatment. H&E staining of the major organs at the time of sacrifice. Scale bar: 100 μm.

Supplementary Table S1

mRNA expression analysis	Forward primers (5'-3');<br>Reverse primers (5'-3')
LAPTM4B	CCTGGATCATCCCATTCTTCTGT<br>AATTAGGAGGCAGTTGCCGTATG
GAPDH	GAAGGTGAAGGTCGGAGTC<br>GAAGATGGTGATGGGATTTC
ATP1A1	GAGCTGCTCTGTGCTTTTCTC<br>TGAAACAGCTGCAGGCTCATA
TRIM8	GAGCGAGAGCAGGACATTGAG<br>CAGTTGGTTCACTTTCTCCTCCA
WWP2	GAGATGGACAACGAGAAG<br>CTCCTCAATGGCATACAG
RNF149	GAGTGACTCTCGGCATGGAG<br>GAGTACCTGCCCCAGTTGTC
NEDD4L	TCTGGAAGGCTGTGCTAC<br>TCTGGGCAGTTTCTCAGG

Supplementary Table S2. Clinical and Pathologic Information of the Patients

Patient NO.	Gender	Histological type	Stage	TNM	Gene Mutation	Biopsies	Age	Treatment	Treatment Time (Month)
1	Female	LUAD	IV	T4N3M1	EGFR exon19 del	Diagnosis	53	Gefitinib	6
						Resistance	53		
2	Female	LUAD	IV-A	T4N2M1a	EGFR exon19 del	Diagnosis	60	Osimertinib	21
						Resistance	62		
3	Female	LUAD	IV	T3N2M1	EGFR exon19 del	Diagnosis	56	Gefitinib	17
						Resistance	58		
4	Female	LUAD	IV	T4N3M1	EGFR exon19 del, TP53 mutation	Diagnosis	52	Almonertinib	8
						Resistance	53		
5	Female	LUAD	IV	T2N3M1c	EGFR exon19 del, TP53 mutation	Diagnosis	49	Osimertinib	16
						Resistance	50		
6	Female	LUAD	IV-A	T2N2M1	EGFR exon19 del	Diagnosis	61	Osimertinib	30
						Resistance	63		

LUAD, lung adenocarcinoma

Supplementary Material and Methods

Reagents, plasmids, antibodies, and siRNAs

The mouse monoclonal anti-Flag (M2) antibody was procured from Sigma-Aldrich (Cat#F1804), while the mouse monoclonal anti-LAPTM4B antibody was sourced from Atlas Antibodies (Cat#AMAb91356). The ATP1A1 antibody was obtained from Proteintech (Cat #55187-1-AP) and Santa Cruz (Cat#sc-71638). The WWP2 antibody (Cat #12197-1-AP), Caspase 3 Polyclonal antibody (Cat #19677-1-AP) , Caspase 7 Polyclonal antibody (Cat #27155-1-AP) and TRIM8 antibody (Cat #27463-1-AP) were acquired from Proteintech. The Ki67 antibody was purchased from Servicebio (Cat#GB111499). EGFR antibody (Proteintech, Cat#66455-1-Ig), p-EGFR antibody (Cell Signaling Technology, Cat #2234S). Antibodies against the following were obtained from Abmart: AKT (Cat#T55561F), p-AKT antibody (Cat#T40067F), HA (Cat#M20003). Antibodies against the following were obtained from ZENBio: ERK (Cat #R24246), p-ERK (Cat #R380698). Anti-Ubiquitin (linkage-specific K63) (Abcam, Cat#Ab179434), K48-linkage Specific Polyubiquitin (Cell Signaling Technology, Cat#8081S). Anti-GFP (Abcam, Cat#Ab290), Anti-mCherry (Abcam, Cat#Ab167453).

Ubiquitin antibody (Proteintech, Cat#10201-2-AP), GAPDH antibody (Proteintech, Cat#60004-1-Ig). The secondary antibodies, Goat Anti-Mouse IgG (H+L)-HRP (Cat#SA00001-1) and Goat Anti-Rat IgG (H+L)-HRP (Cat#SA00001-2), were acquired from Proteintech. Cross-adsorbed Alexa Fluor 488-conjugated Goat Anti-Rabbit IgG (Cat#AS053) and Cy3-conjugated Goat Anti-Mouse IgG (Cat#AS008) were purchased from Abclonal. Protein A/G agarose beads were bought from Santa Cruz (Cat#sc-2003), Anti-DYKDDDDH magnetic agarose were bought from Thermo Scientific (Cat#A36797).

The first siRNAs against ATP1A1 (GATTCGAAATGGTGAGAAA), TRIM8 (CCGCAAGATTCTCGTCTGT), RNF149 (CACCGAACATGTCCAATGT),

WWP2 (CGGACGTGTCTATTATGTT), NEDD4L (GGAGAATTATGTCCGTGAA), the second siRNAs against ATP1A1 (GTCGTCTGATCTTTGATAA), TRIM8 (GTGGACAACTGTTACTGTT), RNF149 (GAGTCTAGCTTTACCAGAT), WWP2 (GGTGCTTCAGCCAGAACAA), and the Control siRNA, were obtained from RIBBIO biotech company.

Western blotting

For detection of total protein, cells were washed with ice-cold phosphate-buffered saline (PBS) and lysed in RIPA lysis buffer (Biosharp, Cat#BL504A), followed by boiling at 98°C for 10 minutes. For detection of phosphorylated protein, cells were scraped in SDS boiling buffer (2.5% SDS, 250 mM Tris/HCl pH 6.8, including 50 mM NaF, 10 mM b-glycerophosphate, 0.5 mM DTT, 0.5 mM PMSF), and lysates were boiled at 95°C for 10 minutes.

Proteins were separated on 8%-12% SDS-PAGE gels and transferred to LF-PVDF membranes (Millipore, Cat#IPVH00010). The membranes were blocked with 3% BSA or 5% non-fat milk in TBST, then incubated with primary antibodies overnight at 4°C. After washing, secondary antibodies were applied, followed by incubation with ultrasensitive ECL chemiluminescent substrate (Biosharp, Cat#BL523B) and imaging using ChemiCapture imaging system (Clinx, Cat#6000Exp). Protein levels were quantified by normalization to the internal control protein (GAPDH) using ImageJ software version 1.53C.

Cell counting kit-8 (CCK8) assay and colony formation experiment

The current study employed a CCK-8 assay and colony formation experiment to assess the drug sensitivity and resistance.

For the CCK-8 assay, 5×10^3 cells were seeded into 96-well plates with 100 µl complete medium, followed by exposure to drugs at specified concentrations for a duration of 48 hours. Subsequent to the treatment, each well received 10 µl of CCK-8 reagent (DOJINDO, Cat#CK04) and were subsequently subjected to a 1-hour incubation. Absorbance measurements at 450 nm were executed using a microplate reader (Spark, TECAN) for quantification.

In the colony formation assay, 3×10^4 cells were seeded into a 12-well plate, cultured at 37°C for a duration of 8 days, with the medium being changed every three days to ensure optimal conditions. Post-culture, cells were fixed utilizing 4% paraformaldehyde, stained with crystal violet, and captured for further analysis.

Immunoprecipitation and Mass-spectrometry

Cellular proteins were extracted using a lysis buffer supplemented with a protease inhibitor cocktail (Beyotime, Cat#P2181S) and incubated on ice for a duration of 30 minutes. Subsequently, the lysate underwent centrifugation at 12,000 rpm for 10 minutes, yielding a supernatant that was subjected to overnight incubation at 4°C on a rotator in the presence of anti-Flag magnetic beads (Beyotime, Cat#P2181S). The ensuing day, the immunoprecipitated complexes underwent a series of three washes with lysis buffer and were subsequently eluted with 3X Flag peptide (Beyotime, Cat#P2181S) at room temperature on a rotator for a period of 120 minutes. Subsequent to a brief 10-second magnetic separation, the resulting supernatant was boiled at 98°C for 10 minutes and subjected to Western blot analysis.

The immunoprecipitated complexes were further analyzed to identify LAPTM4B interaction partners in PC9 cells using mass spectrometry (Shanghai, Bioprofile Biotechnology).

Analysis of ATP1A1 ubiquitination

Cells were cultivated in 60-mm dishes and treated with RIPA lysis buffer (Beyotime, Cat# P0013) supplemented with a protease inhibitor cocktail (MCE, Cat#HY-K0010) before being scraped for harvesting. Prior to collection, a 20 μM MG-132 treatment was administered for 15 hours to impede proteasomal degradation processes. Subsequently, the resulting lysates underwent clarification through centrifugation at 12,000 x g for 10 minutes at 4°C. Equal quantities of the clarified cell lysates were then incubated with 1.6 μg of primary ATP1A1 antibody (Santa Cruz, Cat#sc-71638) for a duration of 3 hours at 4°C on a rocker platform. Following this, 25 μl of protein A/G agarose beads (Santa Cruz, Cat#SC-2003) were introduced, and the mixture was left to incubate overnight at 4°C to enhance the immunoprecipitates.

The resultant immunoprecipitated complexes were subjected to a pair of 10-minute washes utilizing 1 ml RIPA solution to eliminate any nonspecific binding. After discarding the resulting supernatant, the pellet was reconstituted in 50 μl of 1X Sample Buffer and heated at 98°C for 10 minutes. Subsequently, the precipitated proteins were analyzed via Western blotting in order to ascertain the extent of ATP1A1 ubiquitination.

To validate the potential E3 ubiquitin ligases, cells were transfected with siRNAs 72 hours prior to the collection of protein samples. 20 μM MG-132 treatment was administered for 15 hours to impede proteasomal degradation processes. Subsequently, the harvested protein samples underwent immunoprecipitation and Western blotting analysis.

To detect specific types of polyubiquitination, the proteins precipitated after immunoprecipitation were scrutinized through Western blotting using Anti-Ubiquitin (linkage-specific K63) or K48-linkage Specific Polyubiquitin.

Measurement of cell apoptosis by FACS

In this study, the cell apoptosis kit (BestBio, Cat#BB-4101) was utilized for the quantification of apoptosis, following the manufacturer's guidelines. Following a 24-hour cell attachment period, the cells were detached using EDTA-free trypsin, washed twice with pre-cooled PBS, and subsequently resuspended in 400 μL of 1x Annexin-V conjugate solution. A 5 μL volume of Annexin-V-FITC staining solution was introduced, and the cells were incubated for 15 minutes at temperatures ranging between 2-8°C in the absence of light.

Subsequently, 5 μL of PI staining solution was added, and the cells underwent a further incubation period of 5 minutes at 2-8°C in darkness. Subsequently, a minimum of 10,000 cells were collected and subjected to analysis utilizing a flow cytometer sourced from Beckman Coulter (USA).

Immunofluorescence staining and confocal microscopy

For immunofluorescence analysis, cellular fixation was carried out with 4% paraformaldehyde for 25 minutes at ambient temperature. Subsequently, quenching of autofluorescence was achieved by treating the cells with 50 mM NH_4Cl for 10 minutes at room temperature. Following this step, the cells underwent rinsing with Phosphate-Buffered Saline (PBS) and permeabilization using 0.1% Triton X-100 in PBS for 10 minutes. To minimize non-specific interactions, cellular blocking was accomplished by exposure to 10% fetal bovine serum (FBS) in PBS for a duration of 1 hour.

Subsequent to the blocking step, the glass coverslips were incubated with specific primary antibodies, namely anti-Flag (Sigma Cat#F1804, diluted 1:400), and anti-ATP1A1 (Proteintech Cat#55187-1-AP, diluted 1:100), at a temperature of 37°C for a period of 45 minutes. Post-incubation, the cells were meticulously washed with PBS and then exposed to the appropriate secondary antibodies (Abclonal, including Alexa Fluor 488-conjugated Goat Anti-Rabbit

IgG (Cat#AS053) and Cy3 Goat Anti-Mouse IgG (Cat#AS008), at a dilution of 1:200) for 45 minutes at 37°C.

Following the secondary antibody incubation, coverslips were thoroughly washed with PBS, rinsed in MQ-H2O, and mounted onto microscope slides utilizing Anti-fluorescence quenching encapsulant (Biosharp, Cat#BL701A). Subsequently, the acquired images were captured using a Zeiss confocal microscope and subjected to detailed analysis leveraging ImageJ software for subsequent data interpretation.

The isolation of plasma membrane ATP1A1

The isolation was performed utilizing Cell Membrane Protein Extraction Kit (Proteintech, Cat#PK10015), according to the instruction. Briefly, Cells in 100-mm dishes were collected at 4°C, subjected to centrifugation at 500 g for 5 minutes, rinsed twice with cold phosphate-buffered saline (PBS). Subsequently, 1 mL of pre-chilled membrane protein extraction reagent was directly introduced to the cells on ice. A freeze-thaw procedure was repeated twice, alternating between liquid nitrogen and room temperature. The homogenized samples underwent centrifugation at 4°C at 700 g for 10 minutes to gather the supernatant, which was further centrifuged at 4°C at 16,000 g for 30 minutes to induce the precipitation of cell membrane fragments.

Upon the collection of the precipitate, 100 uL of membrane protein extraction reagent was supplemented, leading to a vigorous vortexing process followed by a series of three cycles involving an ice bath. The resulting supernatant, rich in cell membrane proteins, was retrieved through centrifugation at 4°C at 16,000 g for 10 minutes. The cell membrane protein samples were subsequently determined by Western blotting.

Data Mining

TCGA and GTEx database, together with Gene Expression Omnibus (https://www.ncbi.nlm.nih.gov/geo/) were employed to investigate the expression of LAPTM4B or ATP1A1, as well as analyze the relationship between gene expression and cancer patients' survival probability.
